# FgCot1 Regulates Polarized Growth and Conidiation in *Fusarium graminearum* via Gpmk1 MAPK and Tsf1 Transcriptional Pathways

**DOI:** 10.1111/mpp.70321

**Published:** 2026-07-23

**Authors:** Chengliang Li, Shaozhe Qin, Daiyuan Sun, Jingyi Ren, Cong Jiang, Jin‐Rong Xu, Guanghui Wang

**Affiliations:** ^1^ State Key Laboratory for Crop Stress Resistance and High‐Efficiency Production, College of Plant Protection Northwest A&F University Yangling Shaanxi China; ^2^ Department of Botany and Plant Pathology Purdue University West Lafayette Indiana USA

**Keywords:** *Fusarium graminearum*, Gpmk1 pathway, homeobox transcriptional factor, NDR kinase, polarized growth, suppressor mutations

## Abstract

*Fusarium graminearum* causes Fusarium head blight (FHB), a devastating wheat disease that threatens global food security and safety. Nuclear Dbf2‐related (NDR) kinases regulate cellular morphogenesis, but their roles in phytopathogenic fungi remain elusive. Here, we characterized *F. graminearum* NDR kinase FgCot1, whose deletion severely disrupted vegetative growth, conidiation, polarized growth and pathogenicity. Notably, the *Fgcot1* mutant showed genetic instability, producing fast‐growing suppressors with primary (Gpmk1 pathway genes), secondary (MFS transporter *SSF1*) and tertiary (transcription factor *TSF1*) mutations. Functional analyses revealed that disrupting the Gpmk1 pathway partially rescued the *Fgcot1* growth defects due to Gpmk1 hyperactivation, whereas *SSF1* deletion independently enhanced growth. The *TSF1* ^R386C^ mutation suppressed the *Fgcot1* growth defect via a Gpmk1/Ssf1‐independent pathway. Moreover, *FST11* deletion and *TSF1* ^R386C^ mutation significantly alleviated the *Fgcot1* mutant's polarized growth defect and slightly restored conidiation, suggesting that Gpmk1 and Tsf1 function in parallel pathways downstream of FgCot1 kinase. Finally, we demonstrated that FgCot1 interacts with Tsf1 and regulates its protein stability and nuclear accumulation; R386C mutation restored these defects of Tsf1 in the *Fgcot1* mutant. Collectively, FgCot1 kinase integrates Gpmk1 signalling and Tsf1‐dependent transcription to govern polarized growth and conidiation in *F. graminearum*.

## Graphical abstract

FgCot1 is a nuclear Dbf2‐related kinase in *Fusarium graminearum*. It inhibits activation of the Gpmk1 MAPK pathway and interacts with transcription factor Tsf1 to regulate its nuclear localization. FgCot1 integrates Gpmk1 signalling and Tsf1‐dependent transcriptional regulation to govern polarized growth and conidiation in *F. graminearum*.

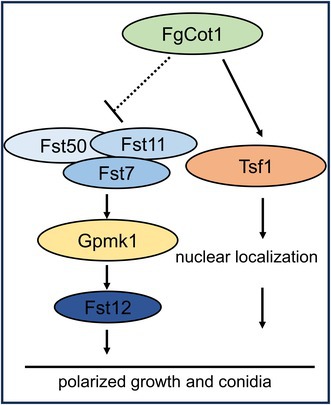

## 1 Introduction

*Fusarium graminearum* is the causal agent of Fusarium head blight (FHB), a globally destructive wheat disease (Bai and Shaner 2004; Goswami and Kistler 2004). Its infection initiates when ascospores land on wheat florets, followed by spore germination and plant cell penetration (Boenisch and Schafer 2011; Jiang et al. 2019). Invasive hyphae then spread to neighbouring spikelets, causing typical FHB symptoms. FHB not only reduces yield but also contaminates grains with mycotoxins such as deoxynivalenol (DON) and zearalenone (ZEN). DON toxin, a potent inhibitor of protein synthesis in eukaryotic organisms, poses severe health risks to humans and animals (De Walle et al. 2010; Audenaert et al. 2013).

Polarized growth is a fundamental biological process driving symmetry breaking and morphogenesis. In fungi, Rho GTPases, such as Cdc42, regulate polarity establishment by interacting with GEFs (guanine nucleotide exchange factors) and GAPs (GTPase‐activating proteins) to recruit polarity‐related proteins, thereby reorganizing actin and microtubule cytoskeletons (Park and Bi 2007). During polarized growth, secretory vesicles are transported to the Spitzenkörper, and are then targeted to specific plasma membrane domains for fusion, supplying enzymes and materials for cell wall expansion (Riquelme 2013). Notably, endocytic recycling is critical for sustaining hyphal apical growth, while vesicles trafficking from synthesis sites to destinations are coordinately regulated by coat proteins, tethering factors, Rab GTPases, motor proteins and SNAREs (Riquelme 2013).

NDR kinases, evolutionarily conserved members of the AGC kinase family, are key regulators of cell polarity and morphogenesis. In fungi, they diverge into two subgroups: *Saccharomyces cerevisiae* Dbf2 and Cbk1, and their *Schizosaccharomyces pombe* orthologs Sid2 and Orb6, which regulate the cell cycle and cell polarity, respectively (Nelson et al. 2003). In *S. cerevisiae* , Cbk1 is essential for cell separation and polarized morphogenesis. The *cbk1* mutants exhibit polarity loss, aberrant morphology, and failed septum degradation between mother and daughter cells, forming multicellular aggregates (Racki et al. 2000; Bidlingmaier et al. 2001). Similarly, *S. pombe orb6* mutants show rounded cell morphology and polarity defects (Verde et al. 1998), highlighting the conserved role of Cbk1/Orb6 kinase in polarity regulation. In filamentous fungi, the NDR kinase Cot1 (an ortholog of Cbk1/Orb6) acts as a master regulator of polarized growth and morphogenesis. In *Neurospora crassa*, the *cot‐1* (ts) mutant displays severely impaired vegetative growth and hyperbranching (Yarden et al. 1992; Ziv et al. 2009). Similar polarity defects are also observed in *cot1* mutants of *Aspergillus nidulans* and *Claviceps purpurea* (Scheffer et al. 2005; Johns et al. 2006), suggesting a conserved role of Cot1 kinase in fungal cell polarity. Cot1 orthologs are critical for pathogenicity in plant pathogens. In *Colletotrichum orbiculare*, CoCbk1 is essential for appressorium morphogenesis and pathogenesis (Kodama et al. 2017), while *C. purpurea* CpCot1 is required for host tissue penetration (Scheffer et al. 2005).

The NDR kinase Cbk1/Orb6/Cot1 is a conserved hub that regulates cell cycle, cell polarity, and morphogenesis. Cbk1 associates with the Ste20‐like kinase Kic1 and Mob2 to form a complex, modulating its kinase activity and substrate specificity (Nelson et al. 2003). In *S. cerevisiae* , Kic1 phosphorylates Cbk1's hydrophobic motif at Thr743, while Mob2 stabilizes the Cbk1 complex to enhance catalytic activity (Jansen et al. 2006; Panozzo et al. 2010). *mob2* and *cbk1* mutants have similar defects including cell cycle arrest and abnormal polarity, confirming their functional interdependence (Colman‐Lerner et al. 2001; Weiss et al. 2002). Structural studies revealed that Mob2 binds to Cbk1's N‐terminal region, stabilizing kinase conformation and facilitating hydrophobic motif phosphorylation. In filamentous fungi, Mob2 orthologs also regulate Cot1 kinase activity (Schmidpeter et al. 2017; Liu et al. 2021), indicating a conserved regulatory mechanism.

Although Cbk1 orthologs play a conserved role in cell polarity across fungi, their downstream regulatory networks exhibit significant divergence. In *S. cerevisiae* , Cbk1 mediates two key pathways to control cellular processes. First, it regulates cell wall remodelling and separation by inhibiting the RNA‐binding protein Ssd1 to derepress the translation of cell wall‐related mRNAs (Jansen et al. 2009; Kurischko et al. 2011). Second, it modulates the localization and activity of transcription factor Ace2 via phosphorylation modification, thereby influencing cell separation, morphogenesis, and gene expression (Nelson et al. 2003). Notably, *S. pombe* Orb6 and *N. crassa* Cot‐1 also regulate mRNA fate by phosphorylating Ssd1 (Nuñez et al. 2016; Herold and Yarden 2017), indicating the regulatory link between Cbk1 orthologs and Ssd1 is conserved in fungi. In contrast, the transcription factor Ace2 is not conserved across the fungal kingdom, being specific to budding yeasts and their close relatives (Maerz and Seiler 2010). It seems that the Cbk1‐Ace2 axis in *S. cerevisiae* is functionally replaced by alternative pathways in other fungal species.

Previously, we identified the FgCot1 kinase in a systematic kinome study of *F. graminearum* (Wang et al. 2011). The deletion of the *FgCOT1* gene caused severe defects in polarized growth, conidiation and pathogenicity. In this study, we found the *Fgcot1* mutant was genetically unstable, readily producing fast‐growing primary, secondary and tertiary suppressors. Sequencing analyses showed that suppressor mutations occurred in Gpmk1 pathway genes (*FST50*, *FST11*, *FST7*, *GPMK1* and *FST12*) and a novel transcriptional factor *TSF1*, suggesting the genetic interaction among FgCot1 kinase, the Gpmk1 signalling pathway and Tsf1‐mediated transcriptional regulation. Subsequently, we demonstrated that FgCot1 kinase functions as a central hub integrating Gpmk1 signalling and Tsf1‐dependent transcription to govern vegetative growth, polarized growth and conidiation.

## 2 Results

### 2.1 FgCot1 Is Important for Polarized Growth and Septum Formation

**FIGURE 1:**
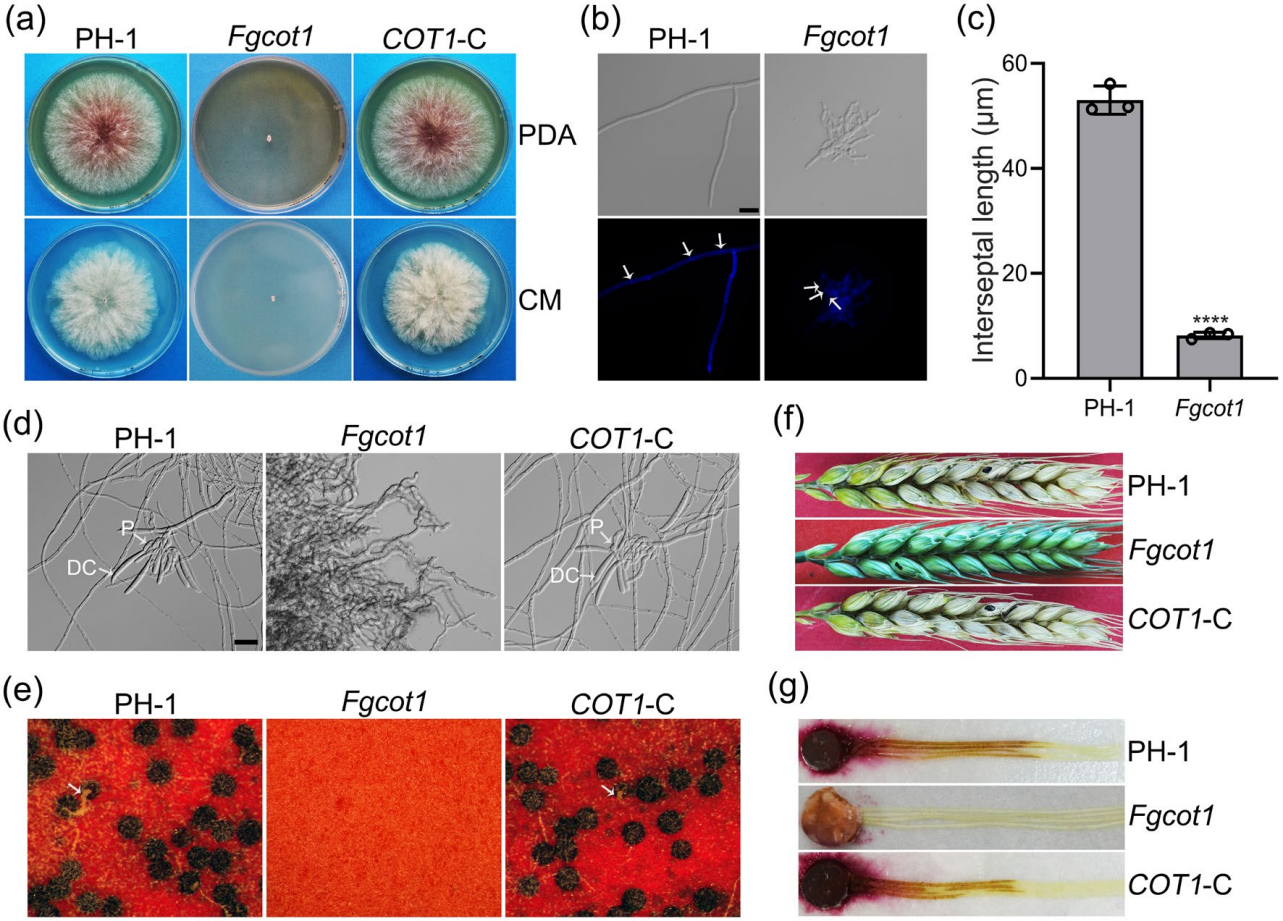
Multiple defects of the *Fgcot1* mutant in vegetative growth, hyphal branching, reproduction and pathogenesis. (a) Colony morphology of the wild‐type strain (PH‐1), *Fgcot1* mutant and complemented transformant (*COT1*‐C) grown on potato dextrose agar (PDA) or complete medium (CM) plate for 3 days. (b) 24‐h‐old hyphae of PH‐1 and *Fgcot1* mutant were stained with Calcofluor White (CFW) and visualized by epifluorescence microscopy. Arrows indicate septa. Bar = 20 μm. (c) The interseptal length in the hyphae of PH‐1 and *Fgcot1* mutant. Mean and standard deviation (SD) were calculated with data from three biological replicates (*n* = 3), each with at least 20 compartments were examined. **** indicates a significant difference determined by Student's *t*‐test (*p* < 0.0001). (d) Phialide formation in 3‐day‐old carboxymethyl cellulose (CMC) cultures of PH‐1 and *COT1*‐C, and in 10‐day‐old CMC cultures of the *Fgcot1* mutant. Arrows indicate phialides, which were absent in the mutant. DC, developing conidium; P, phialide. (e) Perithecium formation of the indicated strains in mating cultures. Arrows point to cirrhi that contains ascospores exuding from perithecia. (f) Wheat heads inoculated with indicated strains and examined at 14 days post‐inoculation (dpi). Inoculation sites marked with black dots. No symptoms observed in *Fgcot1*‐inoculated kernels. (g) Maize silks were inoculated with culture plugs and assessed for discolouration at 5 dpi. For pathogenicity assays, three independent biological replicates were performed, using at least 10 wheat heads or 3 silk bundles per replicate.

The *FgCOT1* (FGSG_01188) gene encodes a 639‐amino acid (aa) protein that contains a Mob‐binding domain (163–255 amino acids [aa]), a protein kinase catalytic domain (260–563 aa), and an AGC‐kinase C‐terminal domain (564–639 aa) (Figure S1a). Its shares 55.09% and 79.66% identity with its orthologs from *S. cerevisiae* and *N. crassa* , respectively (Figure S1b). When grown on potato dextrose agar (PDA) or complete medium (CM), the *Fgcot1* mutant (Table 1) grew extremely slowly (Figure 1a). When grown in yeast extract‐peptone‐dextrose (YEPD) for 24 h, the *Fgcot1* mutant formed wider hyphae and exhibited a hyperbranching phenotype under microscopic observation (Figure 1b), indicating that FgCot1 plays an important role in polarized growth. To determine the potential defect in septation, the *Fgcot1* mutant was stained with Calcofluor White (CFW). When observed under an epifluorescence microscope, the vegetative hyphae of the *Fgcot1* mutant showed denser septa compared to the wild‐type PH‐1 (Figure 1b,c). These results indicate that FgCot1 plays an important role in polarized growth and septum formation in *F. graminearum*.

**TABLE 1:** The wild‐type and mutant strains of *Fusarium graminearum* used in this study.

Strain	Brief description	References
PH‐1	Wild type	Cuomo et al. (2007)
*Fgcot1*	*Fgcot1* deletion mutant	This study
*COT1*‐C	*Fgcot1*/*FgCOT1*‐C complemented transformant	This study
S1–S119	Primary suppressors of Fgcot1 mutant	This study
S1‐1, 2	Secondary suppressors of S1	This study
S23‐1, 2, 3	Secondary suppressors of S23	This study
S27‐1, 2, 3	Secondary suppressors of S27	This study
S47‐1	Secondary suppressor of S47	This study
S52‐1, 2, 3	Secondary suppressors of S52	This study
S71‐1	Secondary suppressor of S71	This study
S52‐1‐3, 7, 8	Tertiary suppressors of S52‐1	This study
*ssf1*‐2, 19	*ssf1* deletion mutants	This study
*SSF1*‐C	*ssf1*/*SSF1*‐C complemented transformant	This study
*tsf1*‐1, 2	*tsf1* deletion mutants	This study
*Fgcot1 fst11*‐5, 6, 7	*Fgcot1 fst11* double mutants	This study
*Fgcot1 gpmk1*‐1,2	*Fgcot1 gpmk1* double mutants	This study
S52 *ssf1*‐1, 2	*ssf1* deletion mutants of S52	This study
*Fgcot1 ssf1*‐1, 2	*Fgcot1 ssf1* double mutants	This study
S52‐1 *TSF* *1* ^R386C^	*TSF1* ^R386C^ mutant of S52‐1	This study
S52‐1 *TSF* *1* ^R386H^	*TSF1* ^R386H^ mutant of S52‐1	This study
S52‐1 *TSF* *1* ^R386L^	*TSF1* ^R386L^ mutant of S52‐1	This study
*TSF1* ^R386C^	*TSF1* ^R386C^ mutant of PH‐1	This study
*TSF1* ^R386H^	*TSF1* ^R386H^ mutant of PH‐1	This study
*TSF1* ^R386L^	*TSF1* ^R386L^ mutant of PH‐1	This study
*Fgcot1* *TSF1* ^R386C^	*Fgcot1* TSF1^R386C^ double mutant	This study
*Fgcot1* *Fst11* *TSF1* ^R386C^	*Fgcot1* *Fst11* *TSF1* ^R386C^ triple mutant	This study
PH‐1/*FgCOT1*‐GFP + *TSF1*‐6His	PH‐1 expressing both *FgCOT1*‐GFP and *TSF1*‐6His constructs	This study
PH‐1/FgCOT1‐GFP	PH‐1 expressing *FgCOT1*‐GFP construct	This study
PH‐1/*TSF1*‐6His	PH‐1 expressing *TSF1*‐6His construct	This study
PH‐1/TSF1‐GFP	PH‐1 expressing *TSF1*‐GFP construct	This study
Fgcot1/TSF1‐GFP	*Fgcot1* expressing *TSF1*‐GFP construct	This study
Fgcot1/TSF1^R386C^‐GFP	*Fgcot1* expressing *TSF1* ^R386C^‐GFP construct	This study

### 2.2 FgCot1 Is Essential for Conidiation, Sexual Reproduction and Pathogenicity

When assayed for conidiation in carboxymethyl cellulose (CMC) medium, the *Fgcot1* mutant failed to produce any conidia at 5 days post‐inoculation (dpi) (Table 2), even after extending the incubation to 14 days. Microscopic examination also revealed that the *Fgcot1* mutant was unable to form phialides (Figure 1d), the specialized structures required for conidiation. In addition, the *Fgcot1* mutant was sterile on mating cultures (Figure 1e), indicating its defect in sexual reproduction. Importantly, in wheat heads infection assays, the *Fgcot1* mutant caused no symptoms on the inoculated kernels and failed to spread to adjacent spikelets (Figure 1f; Table 2), indicating the *Fgcot1* mutant is nonpathogenic. Consistently, the *Fgcot1* mutant could not cause any necrotic lesions in maize silk (Figure 1g). These results indicate that FgCot1 is essential for conidiation, sexual reproduction and pathogenicity.

**TABLE 2:** Phenotypic characterization of *Fgcot1* mutant and its derived suppressors.

Strain	Growth rate (mm/day)^A^	Conidiation (×10^4^/mL)^B^	Disease index^C^
PH‐1	12.56 ± 0.56 a	81 ± 9.81 a	8.5 ± 1.58 a
*Fgcot1*	0.52 ± 0.06 e	0 d	0 b
*COT1*‐C	11.56 ± 0.08 a	80 ± 5.74 a	7.6 ± 2.94 a
S52	1.52 ± 0.08 d	2.0 ± 1.58 c	0 b
S52‐1	2.28 ± 0.08 c	3.0 ± 1.34 c	0 b
S52‐1‐3	3.77 ± 0.13 b	40 ± 8.05 b	0 b
S52‐1‐7	3.59 ± 0.05 b	43 ± 12.84 b	0 b
S52‐1‐8	3.96 ± 0.07 b	43 ± 10.53 b	0 b

*Note:* The means ± SD were calculated from the results of three independent experiments. Statistically significant differences were marked with different letters, determined by one‐way ANOVA followed by Duncan's multiple‐range test (*p* < 0.05).

^A^ The average daily extension of colony radius.

^B^ Conidiation was determined in 5‐day‐old CMC liquid cultures.

^C^ Diseased spikelets per wheat head were counted at 14 days post‐inoculation (dpi).

To further confirm the function of *FgCOT1* gene, the full length of *FgCOT1* gene with its native promoter was re‐introduced into the *Fgcot1* mutant. In the complemented transformant *Fgcot1*/*FgCOT1*‐C (Table 1), all defects including vegetative growth, conidiation, sexual development and pathogenicity were completely restored (Figure 1; Table 2). Therefore, *FgCOT1* is directly responsible for all defects observed in the *Fgcot1* mutant.

### 2.3 The *Fgcot1* Mutant Produces Fast‐Growing Spontaneous Suppressors

**FIGURE 2:**
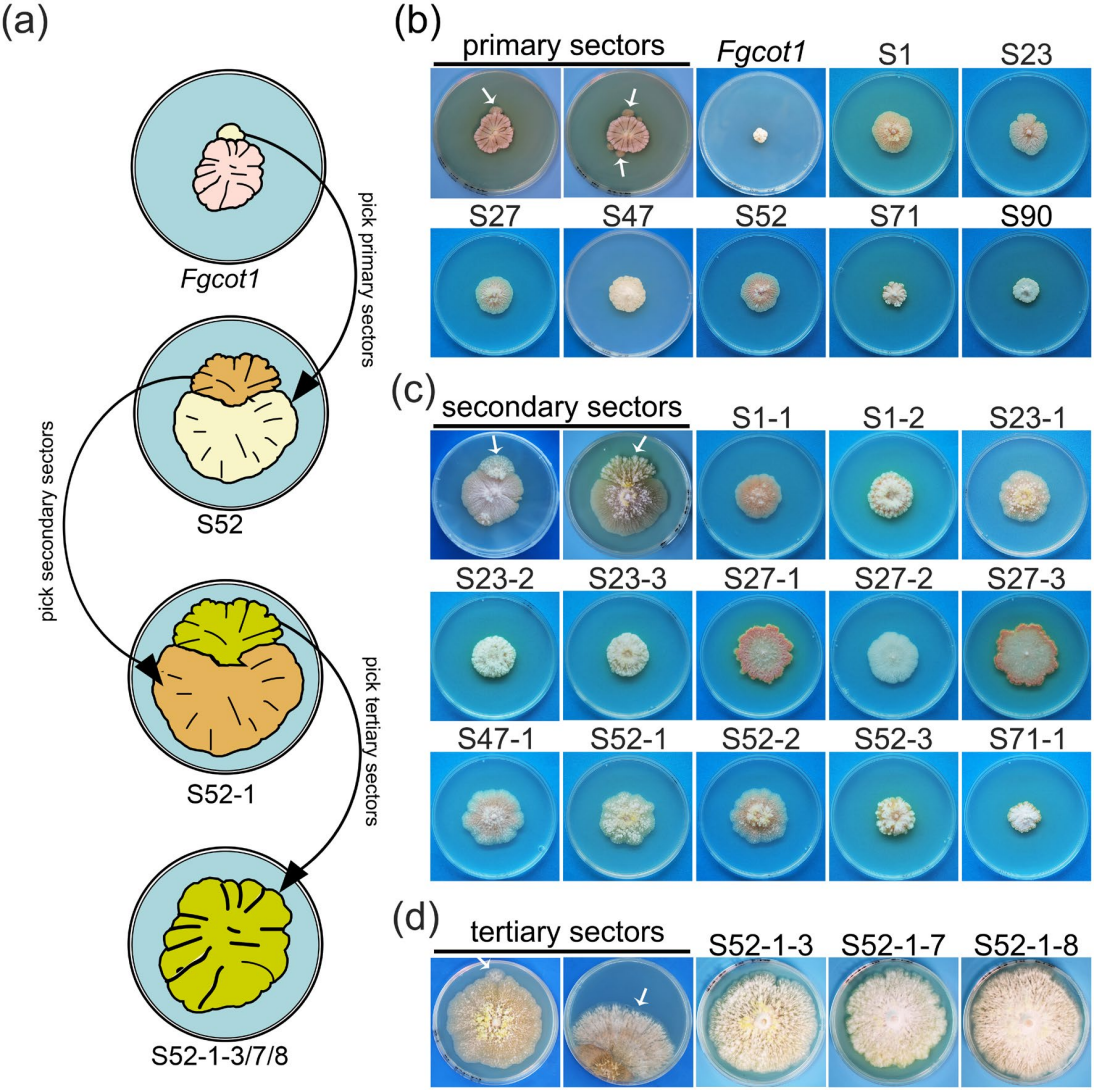
Spontaneous suppressors of the *Fgcot1* mutant. (a) Schematic diagram for picking primary, secondary, and tertiary sectors. (b) Faster‐growing sectors emerging from the margin of the *Fgcot1* mutant after more than 15 days of incubation on potato dextrose agar. The *Fgcot1* mutant along with seven representative primary suppressors were cultured on complete medium (CM) for 10 days. (c) A total of 13 secondary suppressors derived from six primary suppressors (S1, S23, S27, S47, S52 and S71) were grown on CM for 10 days. (d) Three tertiary suppressors (S52‐1‐3, S52‐1‐7 and S52‐1‐8), which were derived from the secondary suppressor S52‐1, were grown on CM (10 days).

The *Fgcot1* mutant exhibited instability, tending to form fast‐growing sectors after incubation on CM plates for 10 days or longer (Figure 2a,b). A total of 119 spontaneous suppressors were obtained from the original *Fgcot1* mutant. Among them, 63 suppressors showed a ≥ 2‐fold increase in growth rate compared to the original *Fgcot1* mutant, yet all remained significantly slower than PH‐1 (Table S2). Seven representative suppressor strains with varying growth rates are shown in Figure 2b. Interestingly, some *Fgcot1* suppressors produced secondary fast‐growing sectors (Figure 2a,c). A total of 13 secondary suppressors were isolated from six primary suppressors (S1, S23, S27, S47, S52 and S71) (Table 1), all of which exhibited significantly faster growth than their corresponding primary suppressors (Figure 2b,c). Notably, we further isolated three tertiary suppressors (S52‐1‐3, S52‐1‐7 and S52‐1‐8) from the secondary suppressor S52‐1 (Figure 2a,d). However, despite exhibiting enhanced growth rates, these three tertiary suppressors, as well as their corresponding secondary suppressor (S52‐1) and primary suppressor (S52), remained sterile on mating cultures and nonpathogenic in wheat head infection assays (Figure S2; Table 2).

**FIGURE 3:**
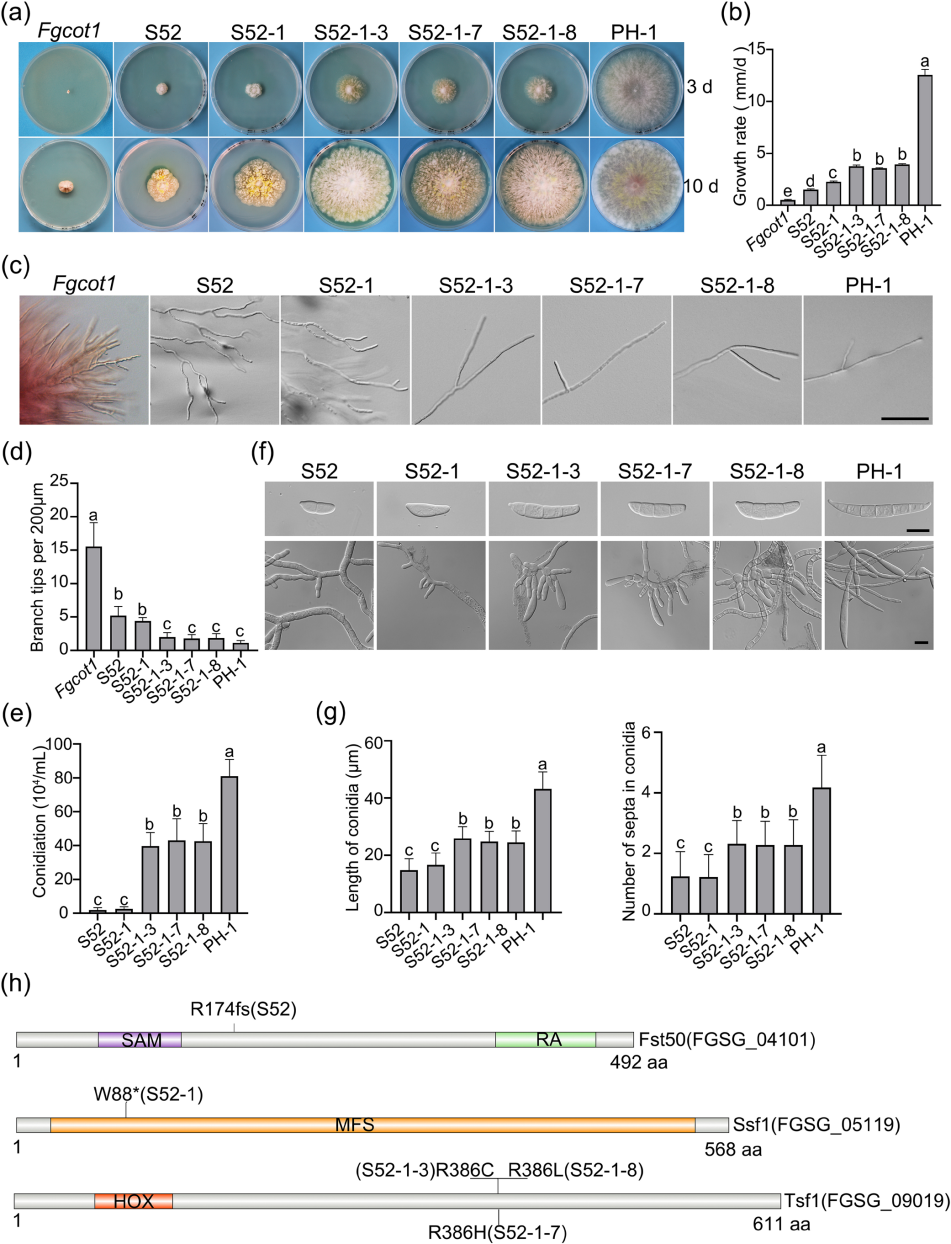
Characterization of the tertiary suppressors and their corresponding secondary and primary suppressors. (a) Colony morphology of the *Fgcot1* mutant, primary suppressor S52, secondary suppressor S52‐1, tertiary suppressors (S52‐1‐3, S52‐1‐7 and S52‐1‐8), and wild‐type strain PH‐1 grown on complete medium (CM) for 3 and 10 days. (b) Growth rates of the indicated strains on CM. Mean and SD were calculated with data from three biological replicates (*n* = 3). (c) Hyphal tip growth and branching patterns at the colony margin. (d) Quantification of branch tips within 200 μm of the hyphal apex for each indicated strain. Data are presented as mean ± SD from three independent biological replicates (*n* = 3), with ≥ 20 hyphae examined per replicate. (e) Conidiation of the indicated strains was measured as conidia per mL in 5‐day‐old carboxymethyl cellulose (CMC) cultures. Mean and SD were calculated with data from three biological replicates (*n* = 3). (f) Phialides and conidia of the indicated strains in 3‐day‐old CMC cultures. (g) Quantitative analysis of septa per conidium and conidial length in the indicated strains. Data are presented as mean ± SD from three independent biological replicates (*n* = 3), with ≥ 50 conidia examined per replicate. (h) Suppressor mutations were identified in *FST50*, *SSF1* and *TSF1* genes by whole‐genome sequencing. Fst50 contains SAM (Sterile alpha motif; InterPro: IPR001660) and RA (Ras association domain; InterPro: IPR000159) domains; Ssf1 harbours an MFS domain (InterPro: IPR020846) with 12 transmembrane domains; Tsf1 possesses a HOX domain (Homeobox domain; InterPro: IPR001356) that typically binds DNA. For (b), (d), (e) and (g), different letters represent significant differences based on one‐way ANOVA followed by Duncan's multiple‐range test (*p* < 0.05).

Further examination showed that the three tertiary suppressors grew at 3.6–4.0 mm/day, approximately 30% of the wild‐type growth rate (12.6 mm/day), and significantly faster than their corresponding secondary suppressor S52‐1 (2.3 mm/day) and primary suppressor S52 (1.5 mm/day) (Figure 3a,b; Table 2), indicating a progressive increase in growth rates from primary to tertiary suppressors. Microscopic analysis further revealed that the *Fgcot1* mutant exhibited denser hyphal branching at the colony edge, whereas this hyperbranching defect was partially alleviated in both primary suppressor S52 and secondary suppressor S52‐1 (Figure 3c,d). Furthermore, the hyperbranching defect was mitigated to an even greater extent in the tertiary suppressors (S52‐1‐3, S52‐1‐7 and S52‐1‐8) (Figure 3c,d). Subsequently, we assayed the conidiation of these suppressor strains. In CMC medium, the *Fgcot1* mutant failed to produce any conidia, while S52 and S52‐1 produced few conidia (2–3 × 10^4^ conidia/mL). Notably, the tertiary suppressors (S52‐1‐3, S52‐1‐7 and S52‐1‐8) produced 40–43 × 10^4^ conidia/mL (> 10‐fold that of S52 and S52‐1), reaching 50% of wild‐type level (Figure 3e; Table 2), with a conidial morphology intermediate between that of S52/S52‐1 and PH‐1 (Figure 3f,g). Consistently, the phialide formation was rarely observed in S52 and S52‐1 but significantly enhanced in the tertiary suppressors (Figure 3f). Therefore, the conidiation defects caused by *FgCOT1* deletion were slightly alleviated in S52 and S52‐1 but significantly mitigated in the tertiary suppressors.

### 2.4 Identification of Mutations in the *Fgcot1* Suppressors

In *F. graminearum*, whole‐genome sequencing has proven to be an efficient strategy for identifying suppressor mutations (Ni et al. 2024). Our results demonstrated that the tertiary suppressors have the greatest potential to suppress the defects of *Fgcot1* mutant (Figure 3a–g). Therefore, the tertiary suppressors (S52‐1‐3, S52‐1‐7 and S52‐1‐8), along with their corresponding secondary (S52‐1) and primary (S52) suppressors, as well as the original *Fgcot1* mutant, were selected for whole‐genome sequencing with over 100× coverage using the Illumina HiSeq‐PE150 platform (Table 3). All mutations in the corresponding genes were confirmed by targeted‐gene sequencing.

**TABLE 3:** Identification of mutations in the *Fgcot1* suppressors by whole‐genome sequencing.

Suppressor strain	Predicted gene	Nucleotide changes	Amino acid changes	Yeast ortholog
S52	FGSG_04101 (*FST50*)	Δ680–713 nt	Arg174fs	Ste50
S52‐1	FGSG_04101 (*FST50*)	Δ680–713 nt	Arg174fs	Ste50
	FGSG_05119 (*SSF1*)	G317A	Trp88*	No ortholog
S52‐1‐3	FGSG_04101 (*FST50*)	Δ680–713 nt	Arg174fs	Ste50
	FGSG_05119 (*SSF1*)	G317A	Trp88*	No ortholog
	FGSG_09019 (*TSF1*)	C1254T	Arg386Cys	No ortholog
S52‐1‐7	FGSG_04101 (*FST50*)	Δ680–713 nt	Arg174fs	Ste50
	FGSG_05119 (*SSF1*)	G317A	Trp88*	No ortholog
	FGSG_09019 (*TSF1*)	G1255A	Arg386His	No ortholog
S52‐1‐8	FGSG_04101 (*FST50*)	Δ680–713 nt		Ste50
	FGSG_05119 (*SSF1*)	G317A	Trp88*	No ortholog
	FGSG_09019 (*TSF1*)	G1255T	Arg386Leu	No ortholog

Abbreviations: *, stop codon; fs, frame shift; Δ, deletion.

Compared to the genome of *Fgcot1* mutant, the primary suppressor S52 contains a frame‐shift mutation (Arg174fs) resulting from a 34‐nucleotide (nt) deletion (680–713 nt) in *FST50* (Table 3), causing 319‐aa C‐terminal truncation of Fst50 (Figure 3h). In secondary suppressor S52‐1, an additional mutation (G317 to A) in FGSG_05119 (a putative MFS transporter) was identified (Table 3), resulting in a nonsense mutation at Trp88 that leads to 481‐aa C‐terminal truncation of FGSG_05119 (Figure 3h). Furthermore, in tertiary suppressors S52‐1‐3, S52‐1‐7 and S52‐1‐8, all harboured mutations in FGSG_09019 (a putative homeobox transcriptional factor), which were absent in both S52 and S52‐1 suppressors (Table 3). Notably, these mutations in FGSG_09019 exclusively occurred at the conserved arginine 386 (R386), resulting in substitutions R386C, R386H and R386L, respectively (Figure 3h).

### 2.5 FgCot1 Kinase Plays a Negative Role in Activating Gpmk1 Pathway

**FIGURE 4:**
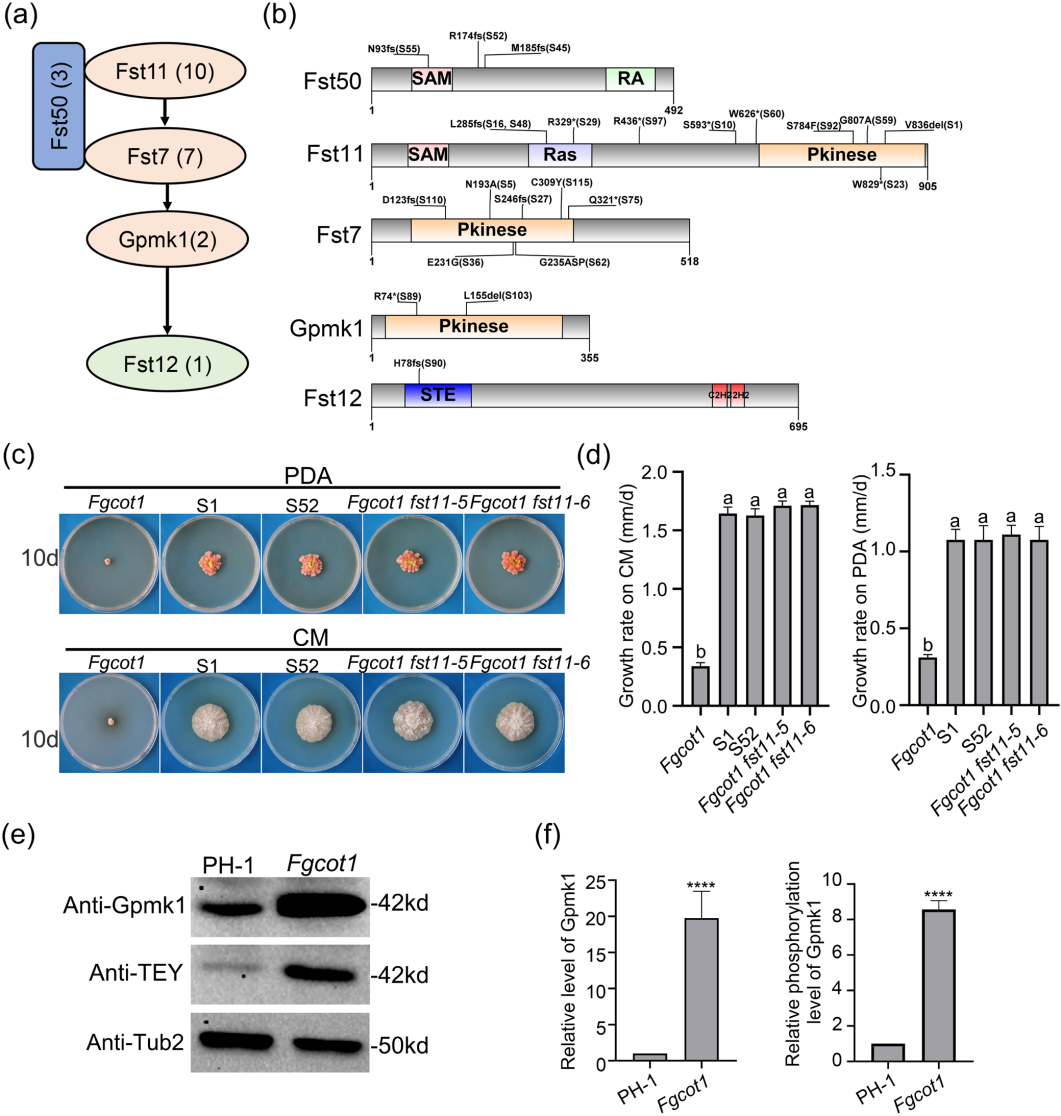
Disruption of Gpmk1 pathway rescues the growth defects in *Fgcot1* mutant. (a) Number of suppressor mutations identified in the Gpmk1 pathway genes (*FST50*, *FST11*, *FST7*, *GPMK1* and *FST12*). (b) Schematic of mutation sites identified in Fst50, Fst11, Fst7, Gpmk1 and Fst12. The asterisk (*) denotes stop codon mutations, whereas ‘fs’ and ‘del’ represent frameshift mutations and deletion mutations, respectively. (c) Ten‐day‐old cultures of the *Fgcot1* mutant, *Fgcot1 fst11* double mutant and suppressor strains S1 and S52. (d) Growth rate of the indicated strains cultured on potato dextrose agar (PDA) and complete medium (CM). Mean and SD were calculated with data from three biological replicates (*n* = 3). Different letters represent significant differences based on one‐way ANOVA followed by Duncan's multiple‐range test (*p* < 0.05). (e) Total proteins isolated from 18‐h vegetative hyphae of the PH‐1 and *Fgcot1* mutant were subjected to western blot, and probed with three antibodies: anti‐TpEY phosphorylation‐specific antibody (for phosphorylated Gpmk1), anti‐Gpmk1 antibody (for total Gpmk1) and anti‐Tub2 antibody (as a loading control). (f) Relative levels of total and phosphorylated Gpmk1 (normalized to the Tub2, with values referenced to the PH‐1). Mean and SD were calculated with data from three biological replicates (*n* = 3). ****Indicates a significant difference determined by Student's *t*‐test (*p* < 0.0001).

Our result showed that the primary suppressor S52 harbours a frameshift mutation (Arg174fs) in the *FST50* gene (Figure 3h, Table 3). In *Magnaporthe oryzae* and *F. graminearum*, the Mst50 ortholog serves as a scaffold protein that facilitates the interaction between Mst11 and Mst7 to activate the Pmk1 pathway (Park et al. 2006; Wang et al. 2011). Similarly, deletion of *MAK‐2* (orthologous to *GPMK1*) partially alleviates the *cot‐1* mutant's defects in *N. crassa* (Maerz et al. 2008). Thus, we hypothesized that disrupting the Gpmk1 pathway might suppress *Fgcot1* mutant's defects. To test this hypothesis, the five Gpmk1 pathway genes (*FST50*, *FST11*, *FST7*, *GPMK1* and *FST12*) were sequenced in 25 primary suppressor strains (Table S3). Whereas two of them had no mutations in these candidate genes, the remaining 23 suppressors exhibited mutations in genes of the Gpmk1 pathway: 3 in *FST50*, 10 in *FST11*, 7 in *FST7*, 2 in *GPMK1* and 1 in *FST12* (Figure 4a,b; Table S3). Notably, over half of these mutations were frameshift or nonsense mutations that are likely to disrupt the Gpmk1 signalling pathway.

In *F. graminearum*, Fst11 activates the Gpmk1 pathway via sequential phosphorylation: it first phosphorylates Fst7, which in turn phosphorylates Gpmk1 (Wang et al. 2011). Among the Gpmk1 pathway genes, *FST11* has the highest mutation frequency (10/25), prompting us to generate the *Fgcot1 fst11* double mutant (Figure S3). This double mutant grew faster than the original *Fgcot1* mutant, phenocopying suppressors S1 and S52 (Figure 4c,d), but remained sterile on mating plates and nonpathogenic in wheat heads (Figure S4). To determine whether suppression was mediated through the Gpmk1 pathway, we generated the *Fgcot1 gpmk1* double mutant, as Gpmk1 is the sole MAPK and terminal kinase of this cascade. Similar to the *Fgcot1 fst11* mutant, the *Fgcot1 gpmk1* mutant exhibited partially restored growth (Figure S5). Therefore, disrupting the Gpmk1 pathway partially rescues the growth defect of the *Fgcot1* mutant.

To clarify the regulatory role of FgCot1 in the Gpmk1 pathway, western blotting with anti‐TpEY, anti‐Gpmk1 and anti‐Tub2 antibodies was performed to assess the total and phosphorylated Gpmk1 in the *Fgcot1* mutant. Interestingly, both total and phosphorylated Gpmk1 levels were significantly increased in the *Fgcot1* mutant compared to the wild‐type PH‐1 (Figure 4e,f). These observations prompted us to examine whether FgCot1 directly regulates the Gpmk1 pathway by phosphorylating upstream components. However, in vitro kinase assays using FgCot1 as the kinase, and Fst11 and Fst7 as candidate substrates detected no phosphorylation of either protein (Figure S6). This result suggests that FgCot1 does not directly phosphorylate Fst11/Fst7, or FgCot1 requires the formation of a protein complex and activation by upstream kinase to exert its activity, as reported for its homologue Cbk1 in *S. cerevisiae* (Jansen et al. 2006; Panozzo et al. 2010). Therefore, loss of FgCot1 enhances Gpmk1 activation, supporting a negative regulatory role for FgCot1 in Gpmk1 signalling, although the underlying molecular mechanism remains to be elucidated.

### 2.6 The Deletion of *SSF1* Enhances the Vegetative Growth Independently of FgCot1 Kinase and Gpmk1 Pathway

**FIGURE 5:**
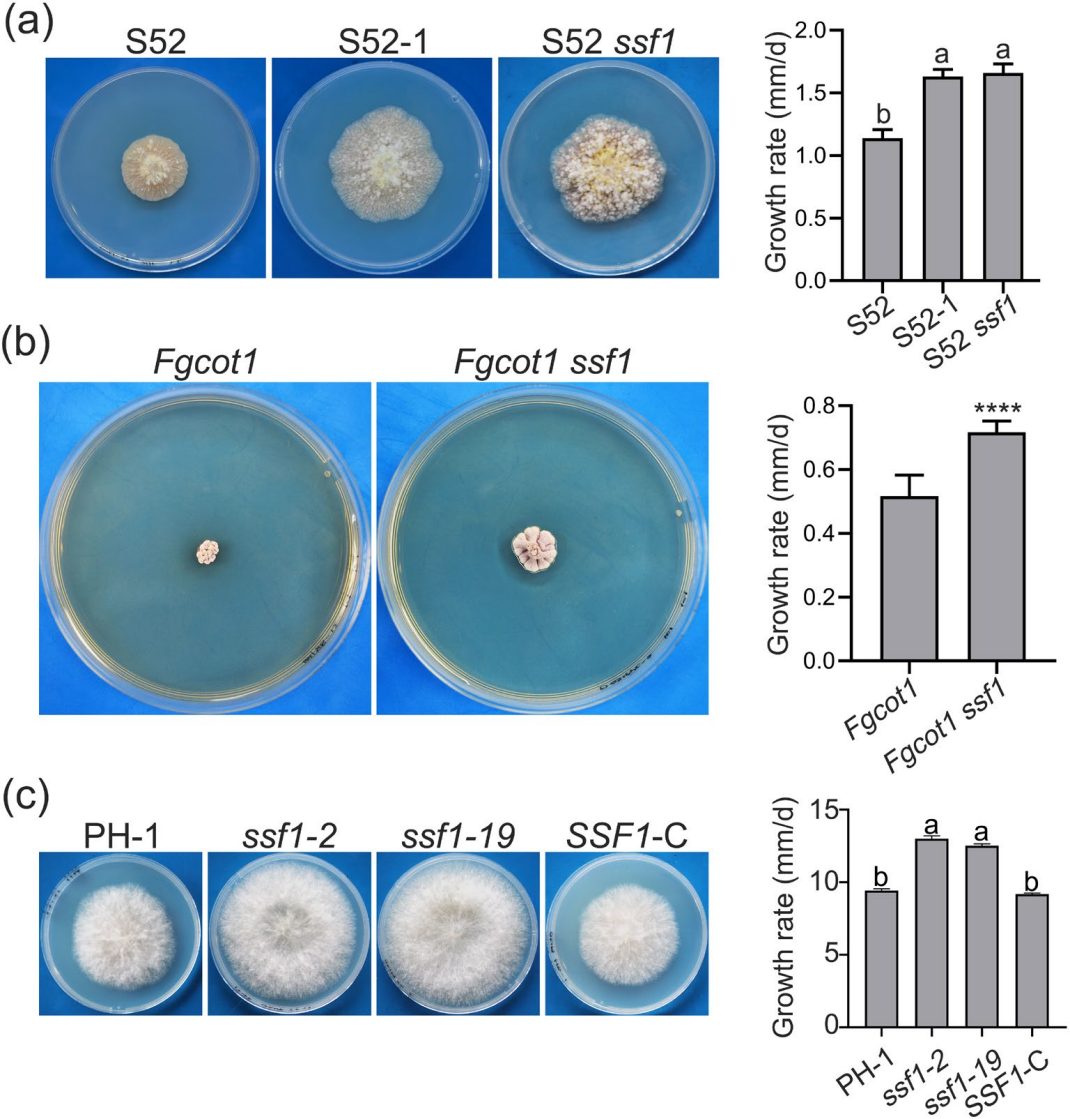
Ssf1 functions as a negative regulator of vegetative growth independent of the Gpmk1 pathway and FgCot1 kinase. (a) Colonies and growth rates of strains S52, S52‐1 and S52 *ssf1* on complete medium (CM) after 10 days. (b) Colonies and growth rates of *Fgcot1* and *Fgcot1 ssf1* mutants on CM after 10 days. (c) Colonies and growth rates of PH‐1, *Fgssf1* and the complemented strain *SSF1*‐C on CM after 3 days. For all bar graphs, mean and SD were calculated with data from three biological replicates (*n* = 3). In (a) and (c), different letters represent significant differences based on one‐way ANOVA followed by Duncan's multiple‐range test (*p* < 0.05). In (b), ****indicates a significant difference determined by Student's *t*‐test (*p* < 0.0001).

In secondary suppressor S52‐1, we identified a mutation (Trp88*) within a putative MSF transporter gene (FGSG_05119) that encodes a protein with 12 transmembrane domains (Figure 3h). In this study, we named this gene as *SSF1*, because it was identified in the secondary suppressor of *Fgcot1* mutant. The Trp88* mutation truncates the 481‐aa C‐terminal region (Figure 3h), probably resulting in a loss of function. To determine the role of *SSF1*, we deleted the *SSF1* in both suppressor S52 and *Fgcot1* mutant (Figure S3c). The resulting S52 *ssf1* mutant grew faster than the suppressor S52, phenocopying the suppressor S52‐1 (Figure 5a). Similarly, the *Fgcot1 ssf1* double mutant exhibited faster growth than the *Fgcot1* mutant (Figure 5b). We also generated *ssf1* single deletion mutants (Figure S3c). Surprisingly, the *ssf1* mutants grew faster than the wild‐type PH‐1 (Figure 5c), indicating that *SSF1* negatively regulates the vegetative growth in *F. graminearum*. These results indicates that *SSF1* deletion enhances the vegetative growth independently of FgCot1 kinase and Gpmk1 signalling pathway in *F. graminearum*.

To further elucidate the function of Ssf1, we performed RNA‐seq analysis to compare global gene expression profiles between the primary suppressor S52 and the secondary suppressor S52‐1 (Table S4). Relative to S52, 405 differentially expressed genes were identified in S52‐1, including 206 upregulated and 199 downregulated genes (Figure S7a). KEGG enrichment analysis showed that these genes were predominantly enriched in metabolic pathways (Figure S7b). Upregulated genes were mainly associated with amino acid metabolism, secondary metabolite biosynthesis and steroid biosynthesis. In contrast, downregulated genes were enriched in glycolysis/gluconeogenesis, secondary metabolite biosynthesis, pyruvate metabolism and carbon metabolism. These findings indicate that Ssf1 plays a central role in maintaining cellular metabolic homeostasis.

### 2.7 FgCot1 Kinase Functions Through Gpmk1 Signalling Pathway and a Distinct Tsf1‐Mediated Pathway

**FIGURE 6:**
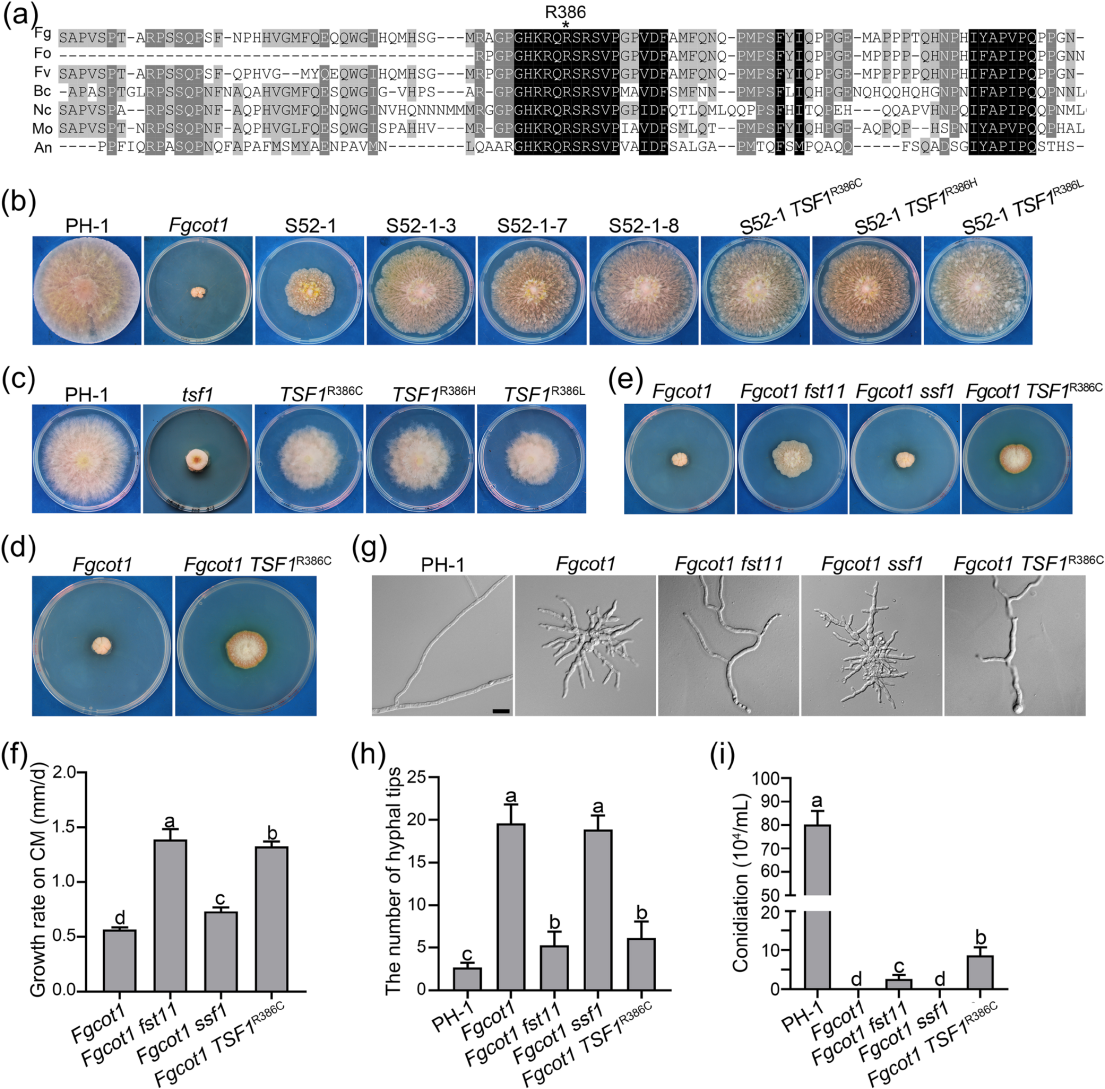
The R386 mutation in *TSF1* can independently restore the phenotypic defects of the *Fgcot1* mutant. (a) Sequencing alignment of Tsf1 region, with the R386 mutation site marked by an asterisk. (b) Ten‐day‐old complete medium (CM) cultures of strains PH‐1, *Fgcot1* mutant, S52‐1, S52‐1‐3/7/8, S52‐1 *TSF1* ^R386C^, S52‐1 *TSF1* ^R386H^ and S52‐1 *TSF1* ^R386L^. (c) Three‐day‐old CM cultures of strains PH‐1, *tsf1*, *TSF1* ^R386C^, *TSF1* ^R386H^ and *TSF1* ^R386L^. (d) Ten‐day‐old CM cultures of *Fgcot1* and *Fgcot1 TSF1* ^R386C^. (e) Ten‐day‐old CM cultures of *Fgcot1*, *Fgcot1 fst11*, *Fgcot1 ssf1* and *Fgcot1 TSF1* ^R386C^. (f) Growth rate of indicated strains on CM plates. Mean and SD were calculated with data from three biological replicates (*n* = 3). (g) The hyphal morphology of indicated strains after 24‐h incubation in TB3 medium. (h) The number of hyphal tips in the same set of strains following 24‐h incubation in TB3 medium. Mean and SD were calculated with data from three biological replicates (*n* = 3), each with at least 20 hyphae examined. (i) The conidial concentrations of indicated strains in 5‐day‐old CMC cultures. Mean and SD were calculated with data from three biological replicates (*n* = 3). For bar graphs in (f), (h) and (i), different letters represent significant differences based on one‐way ANOVA followed by Duncan's multiple‐range test (*p* < 0.05).

In tertiary suppressors (S52‐1‐3, S52‐1‐7 and S52‐1‐8), all mutations were exclusively identified at residue R386 in the FGSG_09019 that encodes a homeobox transcription factor (Figure 3h). In this study, we designated it as *TSF1*, because it was identified in the tertiary suppressor of *Fgcot1* mutant. Sequence alignment revealed that the R386 is located in a conserved region of Tsf1 orthologs from diverse filamentous fungi (Figure 6a), suggesting its critical role in Tsf1 function. However, no ortholog of Tsf1 was identified in budding yeast or fission yeast. When introducing these three R386 mutations individually into suppressor S52‐1 (Figure S8), the resulting transformants S52‐1 *TSF1* ^R386C^, S52‐1 *TSF1* ^R386H^ and S52‐1 *TSF1* ^R386L^ grew faster than S52‐1, phenocopying the tertiary suppressors S52‐1‐3, S52‐1‐7 and S52‐1‐8 (Figure 6b). Thus, the R386C, R386H and R386L mutations in *TSF1* exert equivalent suppressive effects on the growth defect of the S52‐1 suppressor. Moreover, we generated the point mutants (*TSF1* ^R386C^, *TSF1* ^R386H^ and *TSF1* ^R386L^) and *tsf1* single deletion mutant in the wild‐type background (Figure S3; Figure S8). All three R386 mutations resulted in comparable reductions in vegetative growth (Figure 6c), indicating similar effects on vegetative growth. However, the deletion of *TSF1* caused a more severe growth defect than R386 mutations (Figure 6c), suggesting that the R386 mutations do not fully eliminate Tsf1's function. Furthermore, introduction of the *TSF1* ^R386C^ mutation into the wild‐type background significantly reduced pathogenicity (Figure S4), showing that the *TSF1* ^R386C^ mutation also reduces the pathogenicity. To assess whether the *TSF1* ^R386^ mutation's suppressive effect on the *Fgcot1* mutant depends on the Gpmk1 pathway and Ssf1, we generated the *Fgcot1 TSF1* ^R386C^ double mutant (Figure S8). The *Fgcot1 TSF1* ^R386C^ mutant showed faster vegetative growth than the *Fgcot1* mutant (Figure 6d), but was completely defective in sexual reproduction and pathogenicity (Figure S4), indicating the *TSF1* ^R386C^ mutation suppresses *Fgcot1*'s growth defect independently of the Gpmk1 pathway and Ssf1. The R386C mutation probably acts as gain‐of‐function allele, enabling Tsf1 to partially bypass the requirement for FgCot1 kinase.

To further evaluate the relationship between Tsf1 and the Gpmk1 pathway, we generated the *Fgcot1 fst11 TSF1* ^R386C^ triple mutant. This triple mutant grew significantly faster than the *Fgcot1 fst11* and *Fgcot1 TSF1* ^R386C^ double mutants (Figure S9a), suggesting that Tsf1 and the Gpmk1 pathway act through distinct downstream pathways to regulate FgCot1‐dependent growth. Moreover, we examined their transcriptional cross‐regulation by quantifying the *TSF1* expression in the *gpmk1* mutant, as well as *GPMK1*, *FST11* and *FST7* expression in the *tsf1* mutant. Compared with the wild type, all genes exhibited less than 1.4‐fold changes in expression (0.71 < FC < 1.4) (Figure S9b), suggesting no apparent transcriptional cross‐regulation between Tsf1 and the Gpmk1 pathway.

Because deletion of *FST11*, *SSF1* or the *TSF1* ^R386C^ mutation each partially suppressed the *Fgcot1* mutant's growth defect, we compared their suppressive efficiencies in detail. In vegetative growth, the *Fgcot1 fst11*, *Fgcot1 ssf1* and *Fgcot1 TSF1* ^R386C^ mutants grew approximately 145.1%, 29.4% and 134.3% faster than the *Fgcot1* mutant, respectively (Figure 6e,f). Furthermore, *FST11* deletion and *TSF1* ^R386C^ mutation significantly alleviated the hyperbranching defect of the *Fgcot1* mutant, but not the deletion of *SSF1* (Figure 6g,h). For conidiation, both *FST11* deletion and *TSF1* ^*R386C*^ mutation partially restored the conidiation defect in the *Fgcot1* mutant, with conidia reaching approximately 3.2% and 10.8% of the wild‐type level, respectively, but the *SSF1* deletion had no effect (Figure 6i). Therefore, FgCot1 regulates vegetative growth, polarized growth and conidiation through both the Gpmk1 signalling pathway and a distinct Tsf1‐mediated pathway.

### 2.8 The R386C Mutation Promotes the Re‐Accumulation of Tsf1 in Nucleus in *Fgcot1* Mutant by Enhancing Tsf1 Protein Stability

**FIGURE 7:**
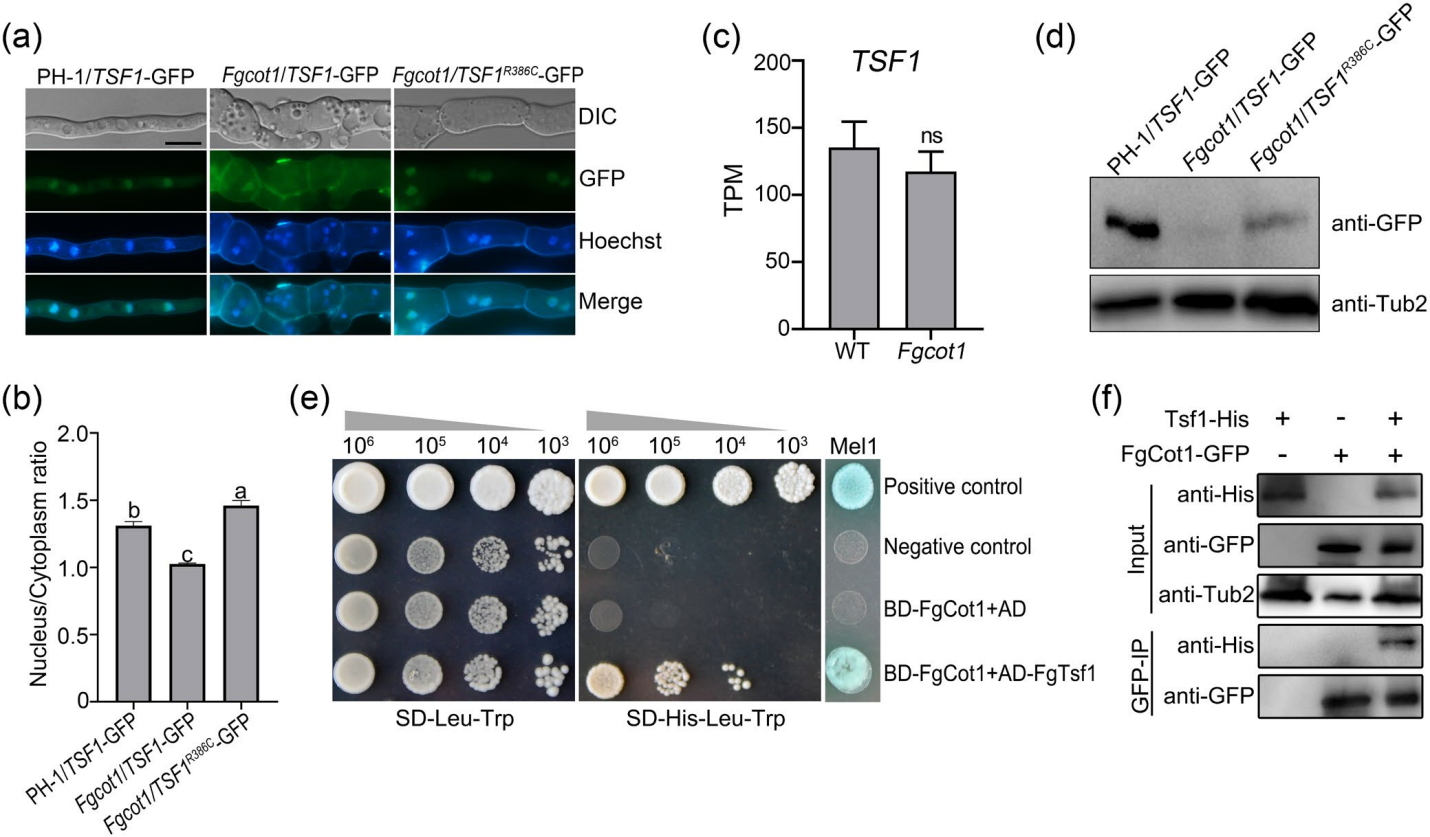
FgCot1 kinase interacts the transcriptional factor Tsf1 and regulates its nuclear localization. (a) Subcellular localization of Tsf1‐GFP and Tsf1^R386C^‐GFP in wild‐type PH‐1 and the *Fgcot1* mutant. Nuclei were stained with Hoechst dye. Bar = 10 μm. (b) The nuclear‐to‐cytoplasmic (N/C) fluorescence intensity ratio was quantified in cells imaged under uniform acquisition parameters. N/C ratios from independent biological replicates were statistically analysed. Data are presented as mean ± SD from three independent biological replicates (*n* = 3), with ≥ 20 cells examined per replicate. Different letters represent significant differences based on one‐way ANOVA followed Duncan's multiple‐range test (*p* < 0.05). (c) The expression levels of *TSF1* from RNA‐seq in PH‐1 and the *Fgcot1* mutant. ns indicates no significant difference determined by Student's *t*‐test. (d) Western blot analysis reveals markedly reduced Tsf1 protein abundance in the *Fgcot1*/*TSF1*‐GFP, which is substantially restored in the *Fgcot1*/*TSF1* ^R386C^‐GFP transformant. (e) Yeast two‐hybrid analysis of the interaction between FgCot1 and Tsf1. Yeast transformants carrying the indicated bait and prey constructs were subjected to serial dilution, then spotted onto SD−Trp−Leu (double dropout, DDO) and SD−Trp−Leu−His (triple dropout, TDO) plates. Expression of the *MEL1* reporter gene was detected via X‐α‐gal staining (blue colour indicates positive expression). (f) Co‐immunoprecipitation (Co‐IP) assays verifying the interaction between FgCot1 and Tsf1.

As a transcription factor, the Tsf1 is predicted to function in the nucleus. To confirm this, we generated a *TSF1*‐GFP fusion construct and transformed it into the PH‐1. In the resulting transformant PH‐1/*TSF1‐*GFP, Tsf1‐GFP was mainly localized to the nucleus, confirming its nuclear localization (Figure 7a,b). To determine whether FgCot1 kinase regulates Tsf1's nuclear accumulation, we introduced the *TSF1*‐GFP construct into the *Fgcot1* mutant. Notably, in the *Fgcot1*/*TSF1*‐GFP transformant, Tsf1‐GFP failed to accumulate in the nucleus (Figure 7a,b), indicating that FgCot1 kinase is essential for Tsf1's nuclear accumulation.

Given that the *TSF1* ^*R386C*^ mutation partially suppressed the *Fgcot1* mutant's defects, we next tested whether it could restore Tsf1's nuclear accumulation in the absence of FgCot1 kinase. Indeed, in the *Fgcot1*/*TSF1* ^R386C^‐GFP transformant, the Tsf1^R386C^‐GFP was successfully relocalized to the nucleus (Figure 7a,b). Therefore, the R386C mutation bypasses the requirement for FgCot1 kinase in promoting Tsf1's nuclear accumulation.

We further examined whether *FgCOT1* deletion affects *TSF1* expression or Tsf1 protein accumulation. The transcriptional level of *TSF1* showed no significant difference between the *Fgcot1* mutant and the wild‐type PH‐1 (Figure 7c). However, the western blot analysis revealed that Tsf1 protein abundance was markedly reduced in the *Fgcot1* mutant (Figure 7d), suggesting that FgCot1 is important for maintaining Tsf1 protein stability. Notably, Tsf1 protein levels were substantially restored in the *Fgcot1*/*TSF1* ^R386C^‐GFP transformant (Figure 7d). Together, these results suggest that the *TSF1* ^R386C^ mutation promotes nuclear re‐accumulation of Tsf1 in the *Fgcot1* mutant, likely by enhancing Tsf1 protein stability.

### 2.9 FgCot1 Physically Interacts With the Transcriptional Factor Tsf1

We assumed that FgCot1 kinase may directly interact with Tsf1 to promote its nuclear accumulation. To test this hypothesis, we employed a yeast two‐hybrid (Y2H) assay to test for a physical interaction between FgCot1 and Tsf1. A bait construct BD‐*FgCOT1* was generated and co‐transformed with a prey construct AD‐*TSF1* into yeast strain AH109. Yeast transformants expressing both *FgCOT1* bait and *TSF1* prey constructs were able to grow on SD−Trp−Leu−His medium and exhibited Mel1 reporter activity, as determined by X‐α‐gal staining (Figure 7e), indicating a direct physical interaction between FgCot1 and Tsf1. To further validate this interaction in vivo, we performed a co‐immunoprecipitation (Co‐IP) assay. The *FgCOT1*‐GFP fusion construct was co‐expressed with the *TSF1*‐His construct in wild‐type PH‐1. In the resulting transformant, the Tsf1‐His protein was successfully detected using an anti‐His antibody in both total proteins and proteins captured by anti‐GFP beads (Figure 7f), further confirming the interaction between FgCot1 and Tsf1. We next performed in vitro kinase assays with FgCot1 and Tsf1, but detected no phosphorylation under the conditions tested (Figure S6). Thus, Tsf1 is not a direct substrate of FgCot1, or FgCot1 alone fails to exhibit kinase activity when expressed in a prokaryotic system.

## 3 Discussion

Our study identified the NDR kinase FgCot1 as a key regulator of vegetative growth, polarized hyphal extension, conidiation, and pathogenicity in *F. graminearum*. Loss of *FgCot1* caused severe growth inhibition, abolished conidiation and virulence, and led to excessive hyphal branching. Analysis of spontaneous suppressors further revealed strong functional connections between FgCot1 and the Gpmk1 MAPK pathway, as well as two additional factors, Ssf1 and Tsf1.

A striking feature of the *Fgcot1* mutant was its tendency to generate fast‐growing suppressors during routine culture. This is probably due to strong selective pressure in the severely compromised *Fgcot1* mutant background, where any secondary mutation that partially restores hyphal growth would be rapidly enriched. The primary suppressor mutations were mainly identified in Gpmk1 pathway genes, including *FST50*, *FST11*, *FST7*, *GPMK1* and *FST12*, indicating a strong genetic interaction between FgCot1 kinase and the Gpmk1 signalling pathway. In *F. graminearum*, the Gpmk1 pathway is indispensable for both sexual reproduction and pathogenicity (Jenczmionka et al. 2003; Urban et al. 2003), which probably explains why primary *Fgcot1* suppressors were not rescued in sexual reproduction and pathogenicity. We further verified that deletion of *FST11* gene could suppress *Fgcot1* mutant's growth defect. Consistently, a previous report showing that deletion of *FST11* or *GPMK1* also alleviates the growth defects of *Fgmob2* mutant, supporting a functional link between the FgCot1–Mob2 complex and the Fst11–Fst7–Gpmk1 module (Liu et al. 2021). Similar relationships have been reported in other fungi. In *N. crassa* , the *cot‐1 mak‐2* double mutant forms dome‐shaped apex, typical of normally growing tips, unlike the growth‐arrested pointed tips of *cot‐1* (ts) mutant (Maerz et al. 2008). Even in *S. cerevisiae* , Cbk1 physically interacts with Ste20 and Ste50, both of which are upstream components of the Kss1/Fus3 pathways (Geyer et al. 1999). Give that Gpmk1 is the ortholog of Mak‐2 and Fus3/Kss1 MAPKs (Wang et al. 2011), the functional association between Cot1/Cbk1‐like kinases and this MAPK module appears to be evolutionarily conserved.

An important question raised by these observations is why inactivation of the Gpmk1 pathway suppresses the growth defects caused by loss of *FgCOT1*. Previous studies in *N. crassa* and *F. graminearum* have shown that *COT1* deletion increases Mak‐2/Mgv1 phosphorylation (CWI pathway) but not Gpmk1 phosphorylation (Maerz et al. 2008; Liu et al. 2021). In *N. crassa* , Cot1 and PKA regulate polarity formation via parallel pathways, acting positively and negatively, respectively (Seiler et al. 2006). The *mak‐2* single mutant or *cot‐1* (ts) *mak‐2* double mutant shows 30%–35% reduced PKA activity (Maerz et al. 2008). Because inhibiting PKA activity suppresses the phenotypic defects of *cot‐1* (ts) (Gorovits and Yarden 2003), *MAK*‐*2* deletion probably suppresses *cot‐1* (ts) defects by reducing PKA activity in *N. crassa* . In this study, however, we showed that both total and phosphorylated Gpmk1 protein levels were significantly increased in the *Fgcot1* mutant, supporting a negative regulatory effect of FgCot1 on the Gpmk1 pathway. Because the phospho‐Gpmk1 signal increased together with total Gpmk1, our data do not distinguish whether FgCot1 primarily affects pathway activation, protein abundance, or both. One possibility is that FgCot1 influences the stability or turnover of Gpmk1 and/or its upstream components. This may also help explain the discrepancy with the previous report, in which phospho‐Gpmk1 was normalized to total Gpmk1 rather than to other internal reference proteins (Liu et al. 2021). Although we did not detect phosphorylation of Fst11 or Fst7 by FgCot1 in vitro, this negative result should be interpreted with caution. Cbk1 kinase typically requires Mob2 co‐factor and activating phosphorylation by an upstream Ste20‐like kinase for full activity (Jansen et al. 2006; Panozzo et al. 2010). Thus, whether FgCot1 regulates the Gpmk1 pathway directly or indirectly remains unresolved.

In addition to the Gpmk1 pathway, our suppressor analysis identified *SSF1* and *TSF1* as two previously unrecognized components associated with FgCot1‐dependent growth regulation. In *N. crassa* or *A. nidulans* , *cot‐1* mutant's defects can be suppressed by multiple mechanisms, including PKA inhibition and osmotic stress (Gorovits and Yarden 2003; Seiler et al. 2006; Shi et al. 2008). Furthermore, a *cot‐1* suppressor mutation was also identified in the *GUL‐1* gene, a yeast *SSD1* ortholog (Terenzi and Reissig 1967; Herold and Yarden 2017). In *S. cerevisiae* , the mRNA‐binding protein Ssd1 suppresses translation of cell wall remodelling proteins to maintain cell integrity under stress and is negatively regulated by Cbk1 (Jansen et al. 2009; Kurischko et al. 2011). In *N. crassa* , the *GUL‐1* deletion partially suppresses *cot‐1* mutant defects by reducing cell wall thickness and inhibiting the overexpression of cell wall remodelling genes (Herold and Yarden 2017). However, the downstream targets of FgCot1 remain poorly characterized in *F. graminearum*. Here, we identified two novel suppressor genes (*SSF1* and *TSF1*) in secondary and tertiary *Fgcot1* suppressors, suggesting that FgCot1 controls additional outputs beyond those described in other systems. The *SSF1* encodes a putative MFS transporter, and its deletion not only rescued the growth defects of the S52 strain (carrying a point mutation in *FST50*) and the *Fgcot1* mutant, but even enhanced the growth of wild‐type PH‐1. Therefore, Ssf1 negatively regulates fungal growth via a mechanism independent of both FgCot1 and Gpmk1. KEGG analysis showed that disruption of *SSF1* remodelled several key metabolic pathways, including secondary metabolism, amino acid biosynthesis, steroid biosynthesis, glycolysis/gluconeogenesis and carbon metabolism. These results suggest that Ssf1 contributes to cellular metabolic homeostasis, probably by mediating the transport of specific substrates. Its disruption may alter the transport and utilization of key nutrients, thereby improving the efficiency of energy and metabolic intermediate use and ultimately promoting hyphal growth.

Among the suppressors, *TSF1* was particularly notable. All three tertiary suppressor mutations occurred at the same residue R386 of Tsf1, which is a homeobox transcriptional factor. The *TSF1* ^R386C^ is a gain‐of‐function allele that can significantly alleviate the defects of both the S52‐1 secondary suppressor and the *Fgcot1* mutant, indicating that its suppressive effect is independent of Gpmk1 and Ssf1. Notably, our results showed that both *FST11* deletion and *TSF1* ^R386C^ mutation partially suppressed the *Fgcot1* mutant defects in vegetative growth, polarized growth and conidiation. Moreover, the combined mutations in both *FST11* and *TSF1* (suppressors S52‐1‐3, 7 and 8) resulted in a greater suppression of *Fgcot1* defects, suggesting that Gpmk1 signalling and Tsf1 contribute to FgCot1‐mediated regulation, although their precise relationship remains unclear. Importantly, however, none of the suppressors restored sexual reproduction or pathogenicity. This probably reflects both the screening regime, which selected only for improved vegetative growth on laboratory medium, and the pleiotropic role of FgCot1 in multiple developmental and pathogenicity‐related processes. Indeed, FgCot1 has also been implicated in cell wall integrity and lipid droplet biogenesis (Liu et al. 2021), both of which are relevant to fungal reproduction and infection. Thus, recovery of these defects may require distinct selective conditions or multiple mutations.

Our data support a close functional link between FgCot1 and Tsf1. As expected for a transcription factor, Tsf1 was predominantly localized in the nucleus in wild‐type PH‐1. In the *Fgcot1* mutant, by contrast, Tsf1 failed to accumulate in the nucleus. The physical interaction between FgCot1 and Tsf1, supported by Y2H and Co‐IP assays, places Tsf1 as a likely downstream effector or closely associated partner of FgCot1. Because arginine is not a phospho‐acceptor residue, R386 is unlikely to represent a direct phosphorylation site. At present, however, our data cannot distinguish whether R386 primarily affects the affinity of the FgCot1–Tsf1 interaction or the intrinsic stability of Tsf1. Instead, this residue may affect Tsf1 conformation, subcellular trafficking or protein stability, thereby altering its dependence on FgCot1. Consistent with this interpretation, *TSF1* transcript levels were unchanged in the *Fgcot1* mutant, whereas Tsf1 protein abundance was markedly reduced, suggesting that FgCot1 regulates Tsf1 post‐transcriptionally. One possibility is that the FgCot1–Tsf1 interaction protects Tsf1 from ubiquitin‐mediated degradation or facilitates its interaction with the nuclear import machinery, thereby promoting Tsf1 stability and nuclear accumulation. Consistent with this model, the *TSF1* ^R386C^ mutation restored the Tsf1 protein stability and nuclear accumulation in the *Fgcot1* mutant, thereby explaining its suppressive effect. A similar regulatory mechanism has been reported in *F. graminearum*, where glycogen synthase kinase Fgk3 regulates FgCreA nuclear localization to modulate cell wall synthesis, whereas H253 deletion promotes the relocalization of FgCreA to the nucleus in the *fgk3* mutant (Ni et al. 2024). Although we did not detect phosphorylation of Tsf1 by FgCot1 in vitro, this does not exclude direct phosphorylation, particularly if the FgCot1 kinase activity depends on additional Mob2 or Kic1 kinase‐mediated phosphorylation as reported in *S. cerevisiae* (Jansen et al. 2006; Panozzo et al. 2010).

Taken together, our results support a model in which FgCot1 coordinates the Gpmk1 MAPK pathway and Tsf1‐associated transcriptional regulation to control vegetative growth, polarized growth, and conidiation in *F. graminearum*. Specifically, FgCot1 negatively influences the Gpmk1 pathway while also promoting Tsf1 accumulation in the nucleus, probably by maintaining Tsf1 protein stability. Our findings reinforce the conserved role of Cot1/Cbk1 family kinases in fungal morphogenesis. Similar polarity defects have been reported in filamentous fungi *N. crassa* , *A. nidulans* and *Trichoderma reesei* (Yarden et al. 1992; Johns et al. 2006; Gao et al. 2020), as well as in the orthologous Cbk1/Orb6 pathways of budding and fission yeasts (Verde et al. 1998; Racki et al. 2000; Bidlingmaier et al. 2001). Future characterization of direct substrates and regulatory partners of FgCot1 will be critical to elucidate how this conserved kinase network modulates fungal development and pathogenicity.

## 4 Experimental Procedures

### 4.1 Fungal Strains and Culture Conditions

The wild‐type PH‐1 (Cuomo et al. 2007) and all mutants generated in this study were routinely cultured on PDA (200 g potato, 20 g glucose and 20 g agar in 1 L water) or CM (10 g glucose, 2 g peptone, 1 g yeast extract, 1 g casamino acids, 6 g NaNO_3_, 0.5 g KCl, 0.5 g MgSO_4_ and 1.5 g KH_2_PO_4_ in 1 L water, pH 6.5) (Ren et al. 2019). Vegetative growth assays were performed with three independent biological replicates. Data are presented as mean ± SD. Statistical significance was determined by one‐way ANOVA followed by Duncan's multiple‐range test (*p* < 0.05) or by Student's *t*‐test, as appropriate. Conidiation was assayed in CMC liquid medium (15 g carboxymethyl cellulose, 1 g yeast extract, 0.5 g MgSO_4_, 1 g NH_4_NO_3_ and 1 g KH_2_PO_4_ in 1 L water), and conidial germination was examined in YEPD (0.3% yeast extract powder, 1% peptone and 2% glucose) as previously described (Wang et al. 2012; Zheng et al. 2012). For sexual reproduction, the aerial hyphae on carrot agar were pressed down with 0.1% Tween 20, and incubated at 25°C under black light (330–400 nm). Perithecium formation was examined at 8 days post‐fertilization (dpf) (Wang et al. 2012). Protoplast preparation and polyethylene glycol (PEG)‐mediated transformation were performed as described (Hou et al. 2002). For transformants selection, the top agar was supplemented with hygromycin B (Coolaber) and G418 (Coolaber) at final concentrations of 300 μg/mL and 400 μg/mL, respectively. Protein was extracted from 18 h germlings harvested from liquid YEPD culture (Ren et al. 2019).

### 4.2 Plant Infection Assays

For wheat head infection assays, 6‐week‐old wheat plants of cultivar Xiaoyan 22 at the flowering stage were used. Mycelial agar plugs from 3‐day‐old PDA cultures of PH‐1 and its derivative mutants were inoculated onto the fifth spikelet from the bottom of each head (with at least 10 wheat heads tested per strain). To maintain a high humidity condition, the inoculated wheat heads were covered and sealed with a plastic bag for 2 days. At 14 days post‐inoculation (dpi), wheat spikelets with typical FHB symptoms were examined and recorded to estimate the disease index as described (Hou et al. 2002). For maize silk infection assays, fungal culture plugs were used as the inoculum, and disease symptom evaluation was conducted at 5 dpi following the previous protocol (Seong et al. 2005). All plant infection assays were independently repeated at least three times.

### 4.3 Generation of the *ssf1*, *Fgcot1 ssf1* and *Fgcot1 fst11* Deletion Mutants

All gene deletion mutants were generated using the split‐marker approach (Catlett et al. 2003). To generate the *ssf1* mutant, a 1‐kb upstream and a 1‐kb downstream flanking sequences of *SSF1* were amplified from the PH‐1 genomic DNA with primer pairs SSF1/1F+2R and SSF1/3F+4R, respectively (Table S1). The resulting PCR products were purified and ligated to the fragments of neomycin resistance gene (*NEO*) amplified from pFL2 plasmid using primer pairs GEN/F+GE/R and GEN/*R*+EN/F, respectively. The resulting PCR products were transformed into the protoplasts of PH‐1. G418‐resistant transformants were screened by PCR with primer pairs G850/F+G852/R, SSF1/5F+6R, SSF1/7F+G855R, and SSF1/8R+G856F. To generate the *Fgcot1 ssf1* mutant, the *SSF1* gene replacement constructs were transformed into *Fgcot1* deletion mutant. The resulting transformants resistant to both hygromycin B and G418 were identified as *Fgcot1 ssf1* double mutant by using PCR analysis. The same split‐marker approach was used to delete the *FgCOT1* gene in the *fst11* mutant background to generate the *Fgcot1 fst11* double mutant. All primers are listed in the Table S1.

### 4.4 Generation of the *TSF1* ^R386C^ , *TSF1* ^R386H^ , *TSF1* ^R386L^ , S52‐1/ *TSF1* ^R386C^ , S52‐1/ *TSF1* ^R386H^ and S52‐1/ *TSF1* ^R386L^ Mutants

A modified split marker approach was used to generate the mutants with in situ mutations (Zhu et al. 2022). The mutated alleles *TSF1* ^R386C^, *TSF1* ^R386H^ and *TSF1* ^R386L^ were amplified from genomic DNA of tertiary suppressors S52‐1‐3, S52‐1‐7 and S52‐1‐8 (harbouring the respective mutations) using primers TSF1site1F and TSF1site2R. The 600‐bp DNA fragment downstream from the terminator sequence of *TSF1* was amplified with primers TSF1site3F‐TSF1site4R. These two fragments were ligated to the *NEO* fragments amplified with primer pairs GEN/F+GE/R and EN/F+GEN/R, respectively, and transformed into PH‐1 to obtain the *TSF1* ^R386C^, *TSF1* ^R386H^ and *TSF1* ^R386L^ mutants. The resulting mutants were further confirmed by sequencing analysis. The same approach was used to generate S52‐1/*TSF1* ^R386H^, S52‐1/*TSF1* ^R386H^ and S52‐1/*TSF1* ^R386L^ mutants in the S52‐1 strain background. All the primers are listed in the Table S1.

### 4.5 Generation of the *FgCOT1* ‐C, *TSF1* ‐GFP and *TSF1* ^R386C^ ‐GFP Constructs

To generate the complemented construct, the full‐length *COT1* with its native promoter and terminator regions was amplified with primers COT/1F+1R, and then co‐transformed with XhoI‐digested pFL2 into yeast strain XK1‐25 as described (Zhou et al. 2011). The complemented construct *FgCOT1*‐C rescued from the resulting Trp^+^ yeast transformants was verified by sequencing analysis and transformed into the *Fgcot1* mutant to obtain the complemented transformant *Fgcot1*/*FgCOT1‐*C. For generating the *TSF1*‐GFP fusion construct, the full‐length *TSF1* gene with its native promoter was amplified with primer pairs pKNTG/TSF1F+TSF1‐R. The PCR product was cloned into the pKNTG plasmid, which had been double‐digested with KpnI and HindIII, using the NovoRec plus One step PCR Cloning kit (Novoprotein). A similar approach was used to generate the *TSF1* ^R386C^‐GFP construct, using genomic DNA extracted from the *TSF1* ^R386C^ mutant as template to amplify the *TSF1* ^R386C^ fragment. Both *TSF1*‐GFP and *TSF1* ^R386C^‐GFP constructs were verified by sequencing, and transformed into PH‐1 and *Fgcot1* mutant, respectively, to obtain transformants PH‐1/*TSF1*‐GFP, *Fgcot1*/*TSF1*‐GFP and *Fgcot1*/*TSF1* ^R386C^‐GFP. All primers are listed in the Table S1.

### 4.6 Spontaneous Suppressors of *Fgcot1* Mutant and Whole‐Genome Sequencing Analysis

Fast‐growing sectors were isolated from the edge of *Fgcot1* mutant colonies grown on PDA plates. To identify mutations in selected suppressors, genomic DNA from 3‐day PDA cultures was sequenced using the Illumina HiSeq‐PE150 platform (Novogene Bioinformatics Institute, Beijing, China) to 100 × coverage with pair‐end libraries. The sequence reads were mapped to the reference genome of wild‐type PH‐1 and the original *Fgcot1* mutant using Bowtie v. 2.23 (Langmead and Salzberg 2012). The suppressor mutation sites were identified by SAMtools with default parameters and annotated by Variant Effect Predictor (VEP) (McLaren et al. 2010). All mutations identified by whole‐genome sequencing analyses were further confirmed by targeted‐gene sequencing. To determine whether other primary *Fgcot1* suppressors carry mutations in Gpmk1 pathway genes (*FST50*, *FST11*, *GPMK1*, *FST7* and *FST12*), the full‐length sequences of these genes were amplified using the corresponding primer pairs and sequenced by Sangon Biotech (Shanghai, China). All primers are listed in the Table S1.

### 4.7 Y2H Assays

To detect the interaction between FgCot1 and Tsf1, the Matchmaker yeast two‐hybrid system (Clontech) was used. The ORF of *FgCOT1* was amplified from first‐strand cDNA of PH‐1 using primer pair BD‐COT1/F+BD‐COT1/R and cloned into bait vector pGBKT7 (BD). Similarly, the ORF of *TSF1* was amplified with primers AD‐TSF1/F+AD‐TSF1/R and inserted into prey vector pGADT7 (AD). After sequencing verification, the bait and prey constructs were co‐transformed into yeast strain AH109 and screened on SD−Leu−Trp plates following the manufacturer's instruction. The transformants isolated from SD−Leu−Trp medium were assayed for growth on SD−Trp−Leu−His medium and Mel1 α‐galactosidase activity using X‐α‐gal. The positive (pGBKT7‐P53 + pGADT7‐T) and negative (pGBKT7‐Lam + pGADT7‐T) controls were provided by the Matchmaker library construction kit (Clontech). For auto‐activation testing, the *FgCOT1* bait construct was co‐transformed with empty pGADT7 vector into yeast strain AH109.

### 4.8 Co‐IP Assays

*FgCOT1*‐GFP fusion construct was generated by cloning the PCR product amplified with primers COT1‐GFP/F + COT1‐GFP/R into RP27‐pKNTG plasmid using the NovoRec plus One step PCR Cloning kit (Novoprotein). The same approach was used to generate the *TSF1*‐6His construct, with *TSF1* amplified with TSF1‐His‐F+TSF1‐His‐R and inserted into RP27‐pKNTG. Sequence‐verified constructs were co‐transformed into wild‐type PH‐1 to generate PH‐1/*FgCOT1*‐GFP+*TSF1*‐6His. As controls, *FgCOT1*‐GFP and *TSF1*‐6His were separately transformed into PH‐1, yielding PH‐1/*FgCOT1*‐GFP and PH‐1/*TSF1*‐6His transformants. The expression of constructs was analysed by PCR and western blot analysis. For Co‐IP assays, total proteins were incubated with anti‐GFP affinity beads and eluted as described (Wang et al. 2011). Total and eluted proteins were detected by western blot using anti‐GFP, anti‐His and anti‐Tub2 (β‐tubulin) antibodies with Enhanced Chemiluminescence (ECL) System (Mishu Biotechnology).

### 4.9 Gpmk1 Phosphorylation Assays

Total proteins were isolated from hyphae of 18 h YEPD cultures. Proteins were separated by a 10% SDS‐PAGE gel and transferred to nitrocellulose membranes (Zhang et al. 2017). Phosphorylated forms of Gpmk1 were detected using the PhophoPlus p44/42 MAPK antibody kit (Cell Signaling Technology) following the manufacturer's instructions. Total Gpmk1 was detected with anti‐Gpmk1 polyclonal antibody (ABclonal) generated against a synthetic peptide (Gpmk1 amino acids 332–347: DFDKHKDNLSKEQLKQ). Immunoreactive bands were quantified with Image Lab software (Bio‐Rad). The Gpmk1 phosphorylation changes were evaluated by relative band densities (phosphorylated vs. total Gpmk1).

### 4.10 RNA‐Seq Analysis

Mycelia of the suppressor strains S52 and S52‐1 were harvested after 3 days of growth on PDA plates. Total RNA was extracted using the Oligotex mRNA Mini Kit (Qiagen). Two independent biological replicates were prepared for each strain. RNA‐seq libraries were constructed and sequenced on an Illumina HiSeq‐2500 platform (2 × 150 bp paired‐end reads) at Novogene Bioinformatics Institute (Beijing, China). The RNA‐seq reads were aligned to the PH‐1 reference genome using Hisat2 (Kim et al. 2015), and featureCounts was used to quantify transcript abundance (Liao et al. 2014). Differentially expressed genes were identified with edgeRun (Dimont et al. 2015). Genes showing a > 2‐fold increase or < 0.5‐fold decrease in expression with *p* < 0.05 were considered differentially expressed.

### 4.11 In Vitro Phosphorylation Assay

To examine the phosphorylation of Tsf1, Fst11 or Fst7 catalysed by FgCot1 kinase, we used prokaryotically expressed and purified recombinant proteins for the reaction. Briefly, 12 μg of purified His‐FgCot1 and 8 μg of GST‐Tsf1 (or GST‐Fst11, GST‐Fst7) were mixed and incubated in kinase buffer (20 mM HEPES pH 7.5, 15 mM MgCl_2_, 1 mM dithiothreitol and 0.5 mM ATP) at 30°C for 60 min. A reaction containing MdRlkt1 kinase and its substrate MdRax2 was included as a positive control (Tang et al. 2026).

The phosphorylated proteins were separated on 7.5% SDS‐PAGE with 50 mM Phos‐tag (ApexBio Technology) and 100 mM MnCl_2_, at 100 V for 1.5 h. Gels were subsequently treated with transfer buffer containing 10 mM EDTA (20 min, three times), washed in transfer buffer for 10 min, and transferred onto methanol‐activated PVDF membranes. His‐FgCot1 and GST‐tagged proteins (Tsf1, Fst11 and Fst7) were detected by immunoblotting using anti‐His and anti‐GST antibodies, respectively. His‐FgCot1, GST‐Tsf1, GST‐Fst11 and GST‐Fst7 were detected by immunoblotting with an anti‐His or anti‐GST antibody, respectively.

## Author Contributions

**Chengliang Li:** writing – original draft, methodology, software, validation, data curation, investigation, visualization. **Shaozhe Qin:** data curation, investigation, validation. **Jin‐Rong Xu:** conceptualization, writing – review and editing, project administration. **Cong Jiang:** conceptualization, writing – review and editing, project administration. **Jingyi Ren:** investigation, validation. **Daiyuan Sun:** investigation, validation. **Guanghui Wang:** conceptualization, funding acquisition, writing – original draft, writing – review and editing, supervision, project administration, resources, methodology, software.

## Funding

This work was supported by grants from the National Natural Science Foundation of China (No. 32270209) and the National Key R&D Program of China (2022YFD1400100).

## Conflicts of Interest

The authors declare no conflicts of interest.

## Supporting information


**Figure S1:** Domain structure and multiple sequence alignment of FgCot1 kinase. (a) Schematic representation of the domains identified in FgCot1 kinase. (b). Sequence alignment of FgCot1 kinase and its orthologs from *Fusarium oxysporum* (Fo), *Trichoderma atroviride* (Ta), *Beauveria bassiana* (Bb), *Verticillium dahliae* (Vd), *Magnaporthe oryzae* (Mo), *Neurospora crassa* (Nc), *Botrytis cinerea* (Bc), *Alternaria alternata* (Aa), *Aspergillus nidulans* (An), *Candida albicans* (Ca), *Saccharomyces cerevisiae* (Sc) and *Schizosaccharomyces pombe* (Sp) via Clustal X 2.1. Identical and similar amino acid residues are shaded in black and grey, respectively.

**Figure S2:** None of the suppressors S52, S52‐1, S52‐1‐3, S52‐1‐7 or S52‐1‐8 rescues the *Fgcot1* mutant's defects in sexual reproduction and pathogenicity. (a) Perithecium formation of indicated strains was examined at 8 days post‐fertilization (dpf). The *Fgcot1* mutant and all suppressor strains failed to produce perithecia (b) Wheat heads inoculated with indicated strains were examined at 14 days post‐inoculation. Inoculation sites marked with black dots. No disease symptoms were observed on kernels inoculated with the *Fgcot1* mutant or any of the suppressor strains. Three independent replicates with at least 10 wheat heads examined in each experiment.

**Figure S3:** The diagnostic PCRs for all the mutants made in this work. (a) Schematic drawing of the primers used to generate gene replacement constructs with *HPH* or *NEO*. (b). PCR verification of *FST11* deletion in the *Fgcot1* background. The mutants *Fgcot1 fst11*‐5, *Fgcot1 fst11*‐6, and *Fgcot1 fst11*‐7 were confirmed using four primer pairs: FST11‐5F/FST11‐6R (L1), G850/G852 (L2), FST11‐7F/G856R (L3) and G855F/FST11‐8R (L4). (c) PCR verification of *SSF1* deletion in the PH‐1, S52, and *Fgcot1* backgrounds. Mutants were confirmed using four primer pairs: SSF1‐5F/SSF1‐6R (L1), G850/G852 (L2), SSF1‐7F/G856R (L3) and G855F/SSF1‐8R (L4). (d) PCR verification of *TSF1* deletion in the PH‐1 background. The mutants *tsf1*‐1 and *tsf1*‐2 were confirmed using four primer pairs: TSF1‐5F/TSF1‐6R (L1), H850/H852 (L2), TSF1‐7F/H856R (L3) and H855F/TSF1‐8R (L4). For each mutant, diagnostic PCR was performed using four primer combinations: target gene‐specific primers (L1), hygromycin resistance gene primers (L2) and primers spanning the 5′ and 3′ junction regions of the gene replacement cassette (L3 and L4).

**Figure S4:** The *Fgcot1 fst11*, *Fgcot1 ssf1* and *Fgcot1 TSF1* ^R386C^ mutants fail to rescue the *Fgcot1* mutant's defects in sexual reproduction and pathogenicity. (a) Conidial morphology of the *Fgcot1 fst11* and *Fgcot1 TSF1* ^R386C^ strains from 5‐day‐old carboxymethyl cellulose (CMC) cultures. (b) Perithecium formation of the indicated strains in mating assays. (c, d) Wheat heads inoculated with indicated strains and photographed at 14 days post‐inoculation.

**Figure S5:** Deletion of *GPMK1* or *FST11* partially alleviates the growth defects in the *Fgcot1* mutant. (a) Ten‐day‐old colonies of the *Fgcot1* mutant, and the *Fgcot1 gpmk1* and *Fgcot1 fst11* double mutants grown on potato dextrose agar (PDA). (b) Growth rates of the indicated strains grown on PDA. Mean and SD were calculated with data from three biological replicates (*n* = 3). Different letters represent significant differences based on one‐way ANOVA followed Duncan's multiple‐range test (*p* < 0.05).

**Figure S6:** In vitro phosphorylation analysis of FgCot1 kinase activity toward Fst11, Fst7 and Tsf1. Recombinant His‐FgCot1 and GST‐tagged substrates (Fst11, Fst7, Tsf1) were expressed in *Escherichia coli* and purified. Purified proteins were incubated in kinase buffer, and phosphorylation was detected by Phos‐tag SDS‐PAGE. The MdRlkt1 kinase and its substrate MdRax2 served as a positive control.

**Figure S7:** RNA‐seq analysis of S52 and S52‐1 suppressor strains. (a) Volcano plot showing 206 genes significantly upregulated (red dots) and 199 genes significantly downregulated (green dots) in S52‐1 relative to S52. (b) Top 15 KEGG pathways enriched among the significantly upregulated and downregulated genes, respectively.

**Figure S8:** Generation and sequence verification of the *TSF1* ^R386C/H/L^ in situ mutations. (a) Schematic diagram of the primers used to introduce the point mutations. (b) Sequencing analysis confirming the S52 *TSF1* ^R386C^, *TSF1* ^R386C^, *Fgcot1 TSF1* ^R386C^, S52 *TSF1* ^R386H^, *TSF1* ^R386H^, S52 *TSF1* ^R386L^, *TSF1* ^R386L^ mutants.

**Figure S9:** The *Fgcot1 fst11 TSF1* ^R386C^ triple mutant exhibits enhanced growth and no apparent transcriptional cross‐regulation between Tsf1 and the Gpmk1 pathway. (a) Colony morphology of the *Fgcot1 TSF1* ^R386C^, *Fgcot1 fst11* and *Fgcot1 fst11 TSF1* ^R386C^ mutants grown on complete medium (CM) for 10 days. (b). Relative expression of *TSF1* in the *gpmk1* mutant. (c). Relative expression of *GPMK1*, *FST11* and *FST7* in the *tsf1* mutant. All expression changes remained within 1.4‐fold of wild‐type levels (0.71 < fold change < 1.4), indicating no apparent transcriptional cross‐regulation.

**Table S1:** Polymerase chain reaction (PCR) primers used in this study.

**Table S2:** Relative growth rates of suppressor strains compared with the original *Fgcot1* mutant.

**Table S3:** Candidate Gpmk1 pathway genes sequenced in the selected suppressor strains.

**Table S4:** Differentially expressed genes (DEGs) up‐ and downregulated in the S52‐1 compared with S52.

## Data Availability

The data that support the findings of this study are available from the corresponding author upon reasonable request.
