# Supplementary figures and images for: FgCot1 Regulates Polarized Growth and Conidiation in Fusarium graminearum via Gpmk1 MAPK and Tsf1 Transcriptional Pathways
Source: Mol Plant Pathol. 2026 Jul 23;27(7):e70321. doi: 10.1111/mpp.70321 (PMC13396691; doi:10.1111/mpp.70321)

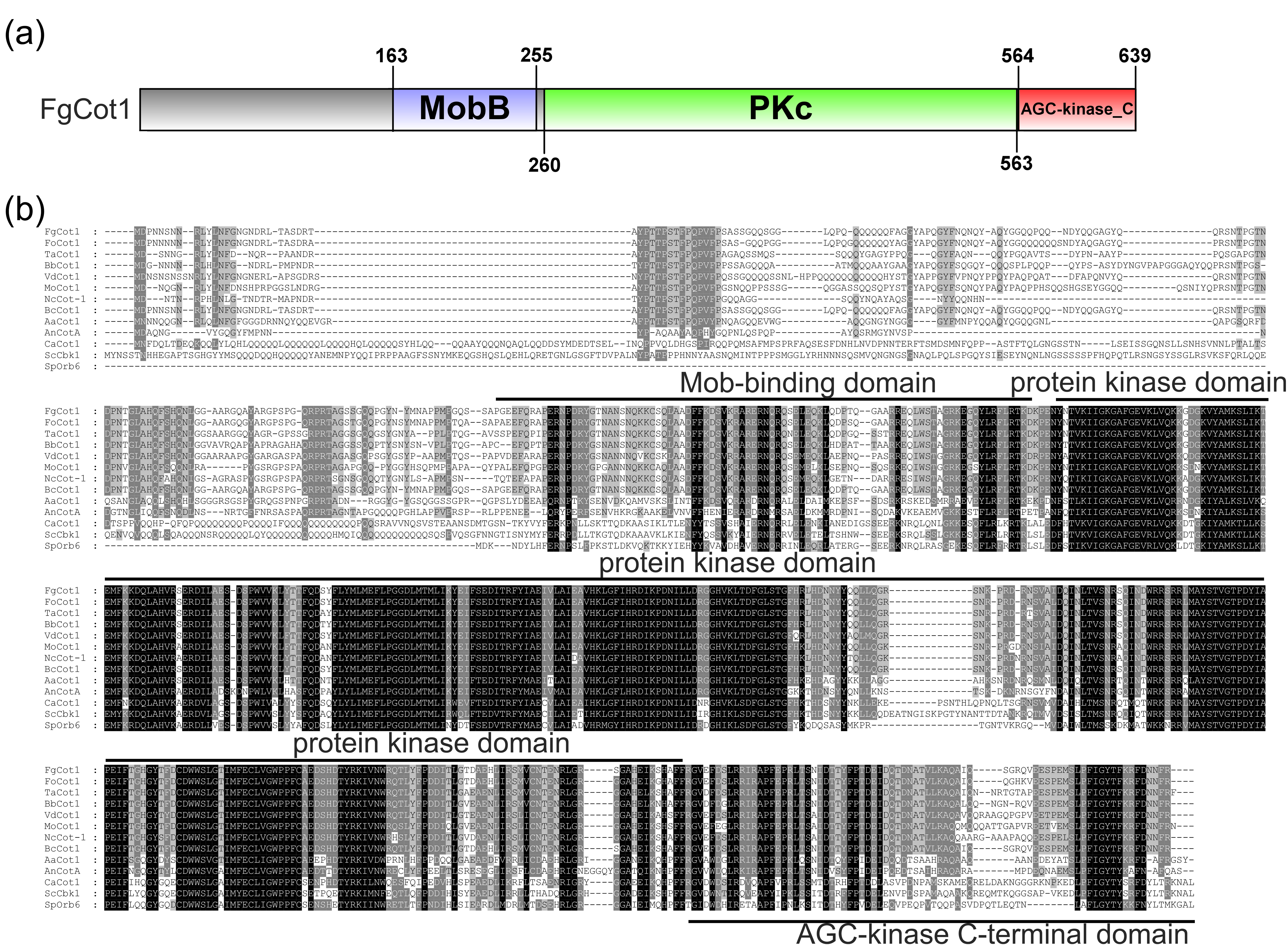

Supplement: Supplementary file 1 — Figure S1: Domain structure and multiple sequence alignment of FgCot1 kinase. (a) Schematic representation of the domains identified in FgCot1 kinase. (b). Sequence alignment of FgCot1 kinase and its orthologs from Fusarium oxysporum (Fo), Trichoderma atroviride (Ta), Beauveria bassiana (Bb), Verticillium dahliae (Vd), Magnaporthe oryzae (Mo), Neurospora crassa (Nc), Botrytis cinerea (Bc), Alternaria alternata (Aa), Aspergillus nidulans (An), Candida albicans (Ca), Saccharomyces cerevisiae (Sc) and Schizosaccharomyces pombe (Sp) via Clustal X 2.1. Identical and similar amino acid residues are shaded in black and grey, respectively. [file MPP-27-e70321-s011.jpg]

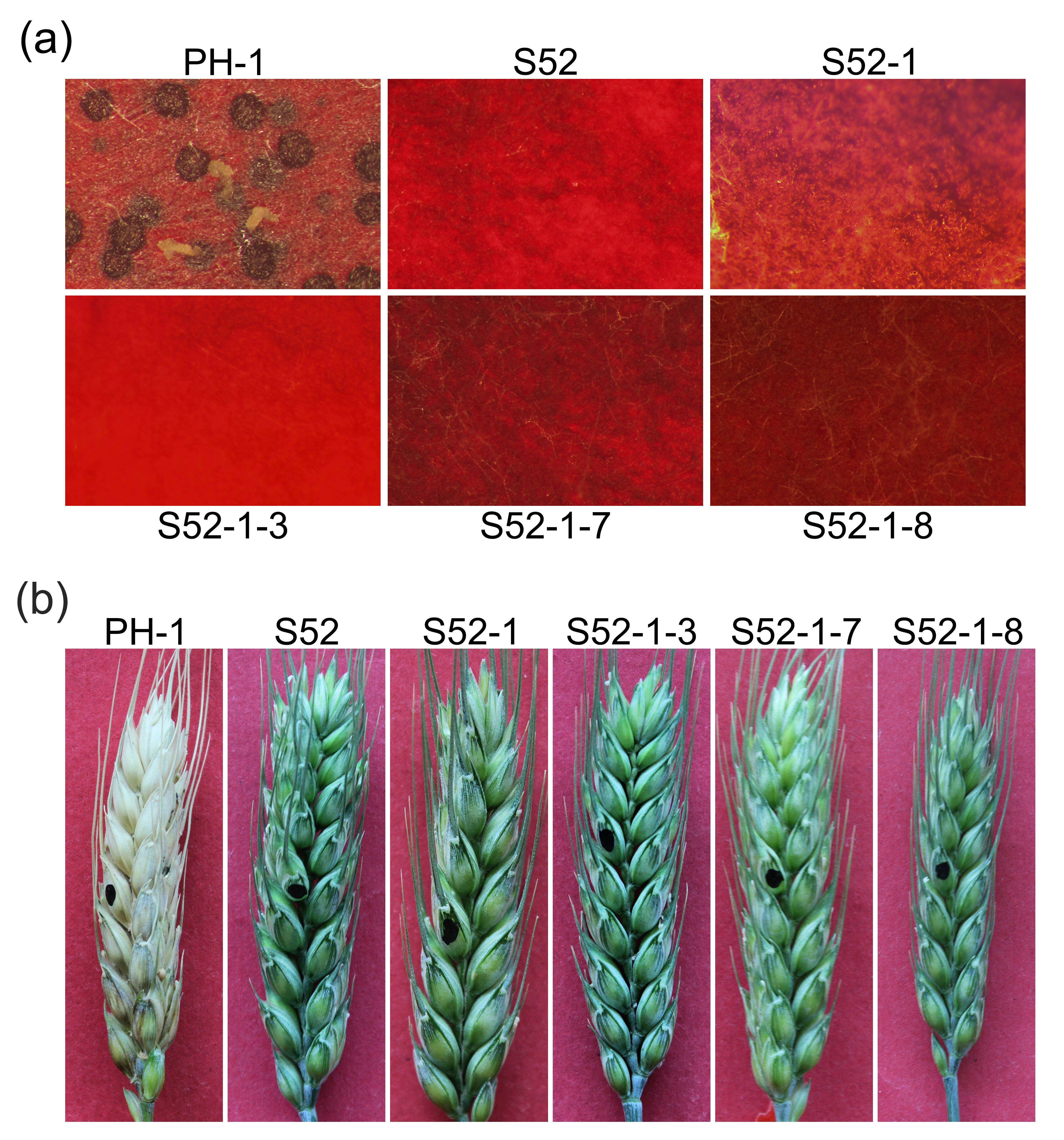

Supplement: Supplementary file 2 — Figure S2: None of the suppressors S52, S52‐1, S52‐1‐3, S52‐1‐7 or S52‐1‐8 rescues the Fgcot1 mutant's defects in sexual reproduction and pathogenicity. (a) Perithecium formation of indicated strains was examined at 8 days post‐fertilization (dpf). The Fgcot1 mutant and all suppressor strains failed to produce perithecia (b) Wheat heads inoculated with indicated strains were examined at 14 days post‐inoculation. Inoculation sites marked with black dots. No disease symptoms were observed on kernels inoculated with the Fgcot1 mutant or any of the suppressor strains. Three independent replicates with at least 10 wheat heads examined in each experiment. [file MPP-27-e70321-s001.jpg]

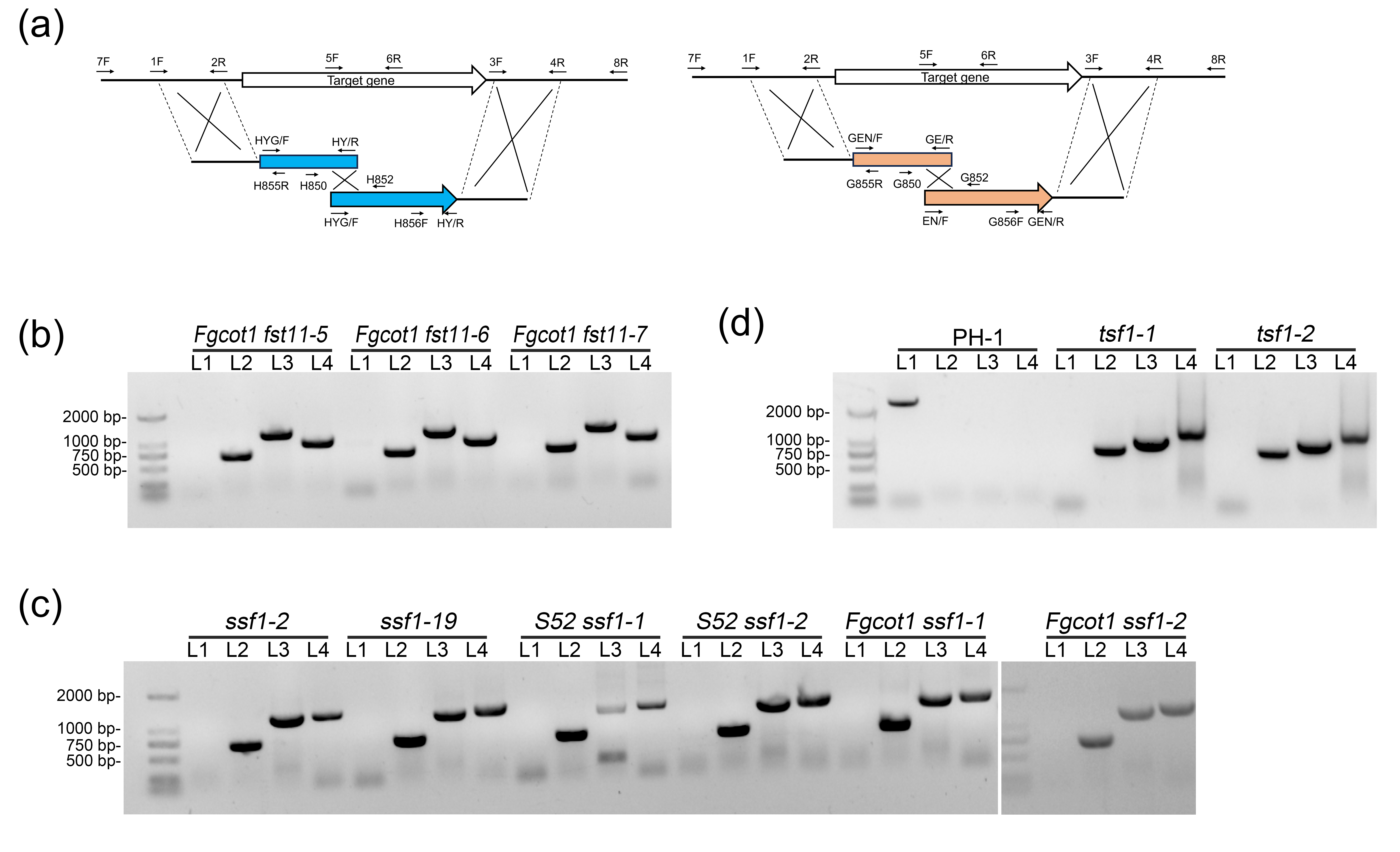

Supplement: Supplementary file 3 — Figure S3: The diagnostic PCRs for all the mutants made in this work. (a) Schematic drawing of the primers used to generate gene replacement constructs with HPH or NEO. (b). PCR verification of FST11 deletion in the Fgcot1 background. The mutants Fgcot1 fst11‐5, Fgcot1 fst11‐6, and Fgcot1 fst11‐7 were confirmed using four primer pairs: FST11‐5F/FST11‐6R (L1), G850/G852 (L2), FST11‐7F/G856R (L3) and G855F/FST11‐8R (L4). (c) PCR verification of SSF1 deletion in the PH‐1, S52, and Fgcot1 backgrounds. Mutants were confirmed using four primer pairs: SSF1‐5F/SSF1‐6R (L1), G850/G852 (L2), SSF1‐7F/G856R (L3) and G855F/SSF1‐8R (L4). (d) PCR verification of TSF1 deletion in the PH‐1 background. The mutants tsf1‐1 and tsf1‐2 were confirmed using four primer pairs: TSF1‐5F/TSF1‐6R (L1), H850/H852 (L2), TSF1‐7F/H856R (L3) and H855F/TSF1‐8R (L4). For each mutant, diagnostic PCR was performed using four primer combinations: target gene‐specific primers (L1), hygromycin resistance gene primers (L2) and primers spanning the 5′ and 3′ junction regions of the gene replacement cassette (L3 and L4). [file MPP-27-e70321-s005.jpg]

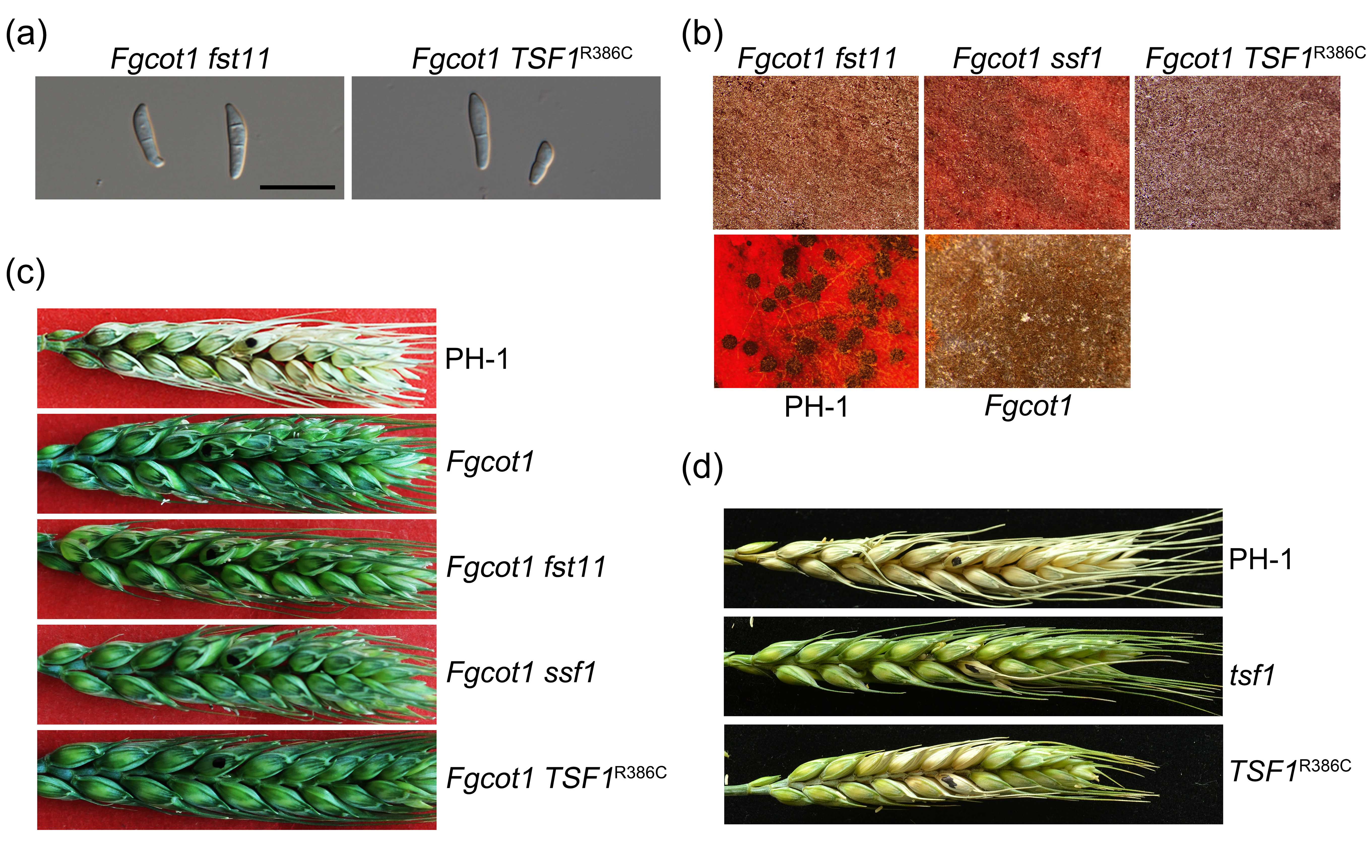

Supplement: Supplementary file 4 — Figure S4: The Fgcot1 fst11, Fgcot1 ssf1 and Fgcot1 TSF1 R386C mutants fail to rescue the Fgcot1 mutant's defects in sexual reproduction and pathogenicity. (a) Conidial morphology of the Fgcot1 fst11 and Fgcot1 TSF1 R386C strains from 5‐day‐old carboxymethyl cellulose (CMC) cultures. (b) Perithecium formation of the indicated strains in mating assays. (c, d) Wheat heads inoculated with indicated strains and photographed at 14 days post‐inoculation. [file MPP-27-e70321-s003.jpg]

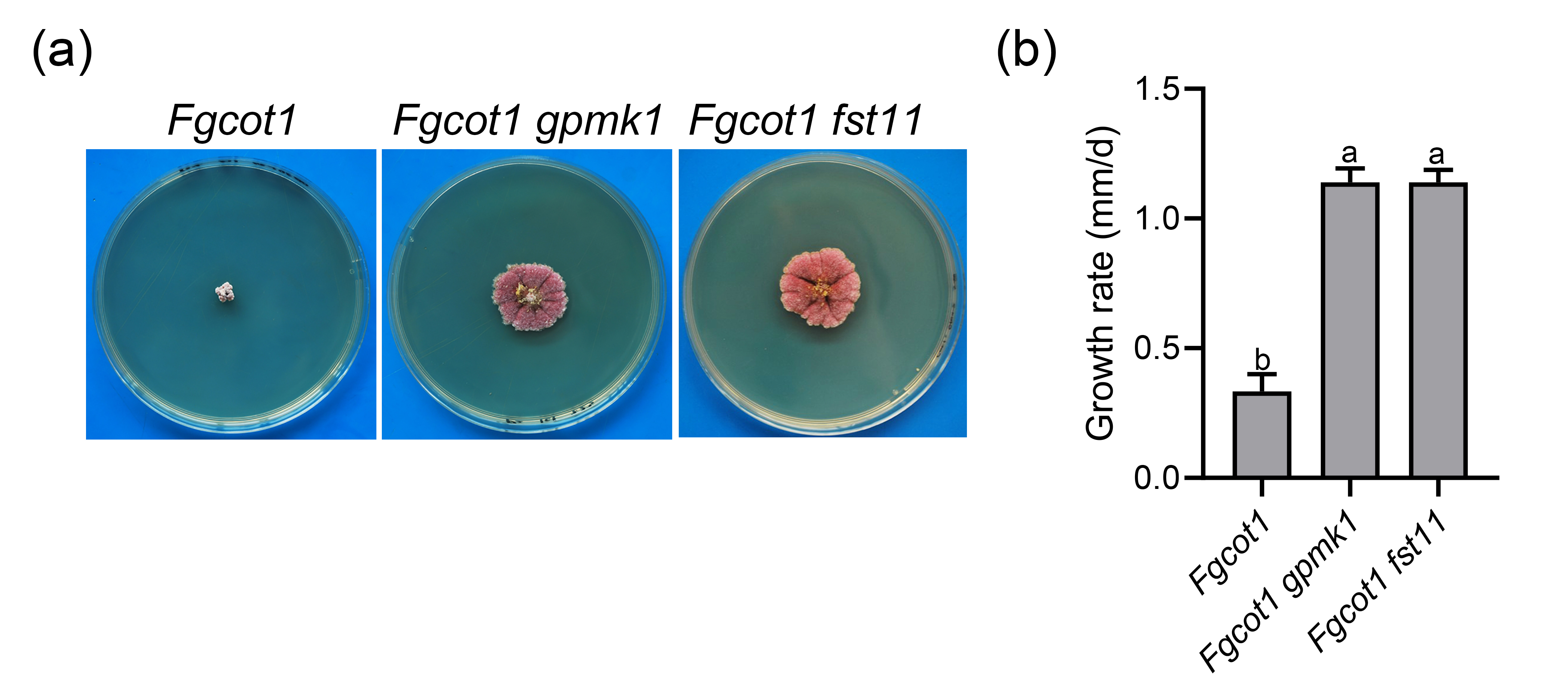

Supplement: Supplementary file 5 — Figure S5: Deletion of GPMK1 or FST11 partially alleviates the growth defects in the Fgcot1 mutant. (a) Ten‐day‐old colonies of the Fgcot1 mutant, and the Fgcot1 gpmk1 and Fgcot1 fst11 double mutants grown on potato dextrose agar (PDA). (b) Growth rates of the indicated strains grown on PDA. Mean and SD were calculated with data from three biological replicates (n = 3). Different letters represent significant differences based on one‐way ANOVA followed Duncan's multiple‐range test (p < 0.05). [file MPP-27-e70321-s008.jpg]

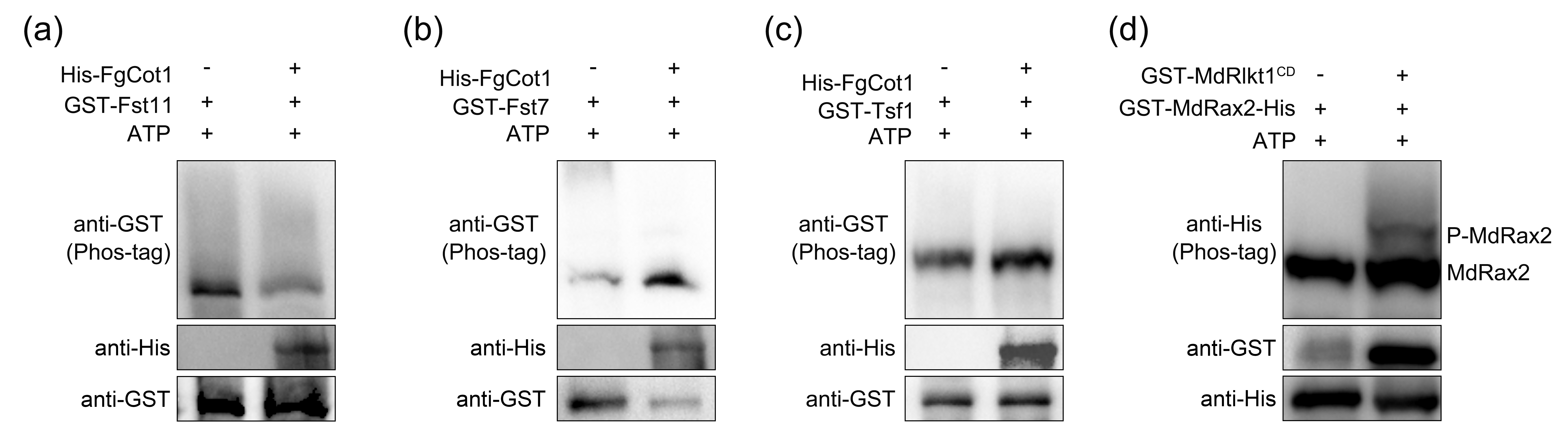

Supplement: Supplementary file 6 — Figure S6: In vitro phosphorylation analysis of FgCot1 kinase activity toward Fst11, Fst7 and Tsf1. Recombinant His‐FgCot1 and GST‐tagged substrates (Fst11, Fst7, Tsf1) were expressed in Escherichia coli and purified. Purified proteins were incubated in kinase buffer, and phosphorylation was detected by Phos‐tag SDS‐PAGE. The MdRlkt1 kinase and its substrate MdRax2 served as a positive control. [file MPP-27-e70321-s009.jpg]

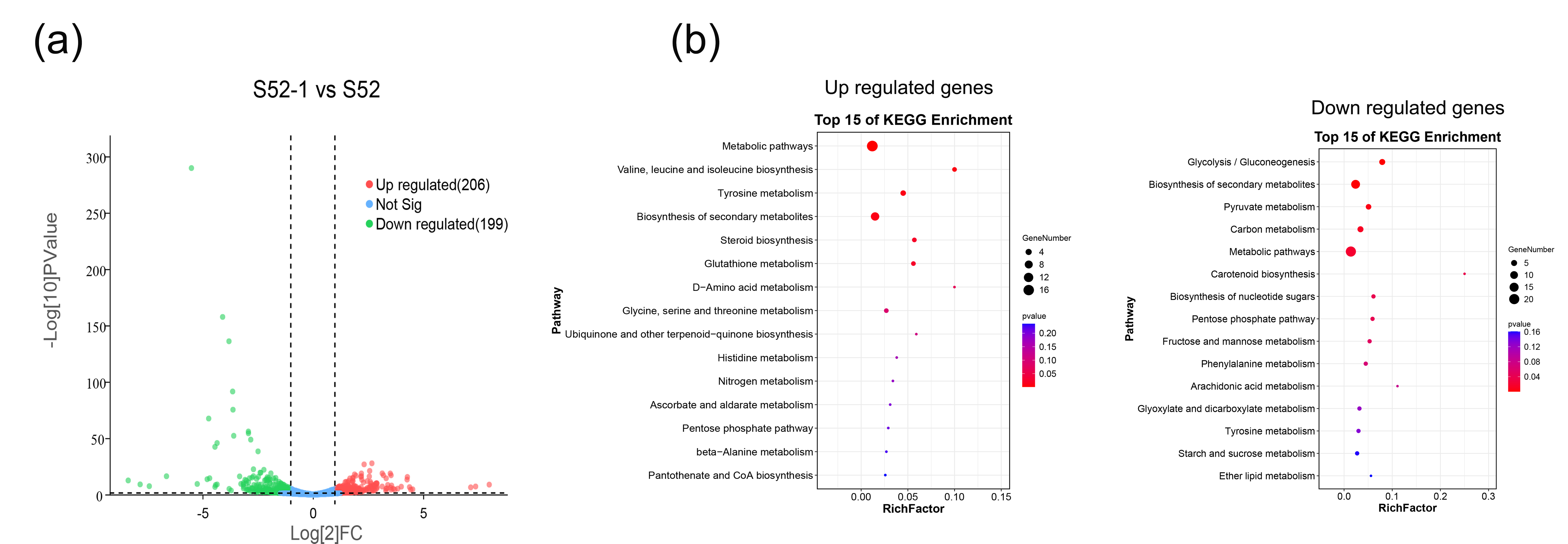

Supplement: Supplementary file 7 — Figure S7: RNA‐seq analysis of S52 and S52‐1 suppressor strains. (a) Volcano plot showing 206 genes significantly upregulated (red dots) and 199 genes significantly downregulated (green dots) in S52‐1 relative to S52. (b) Top 15 KEGG pathways enriched among the significantly upregulated and downregulated genes, respectively. [file MPP-27-e70321-s004.jpg]

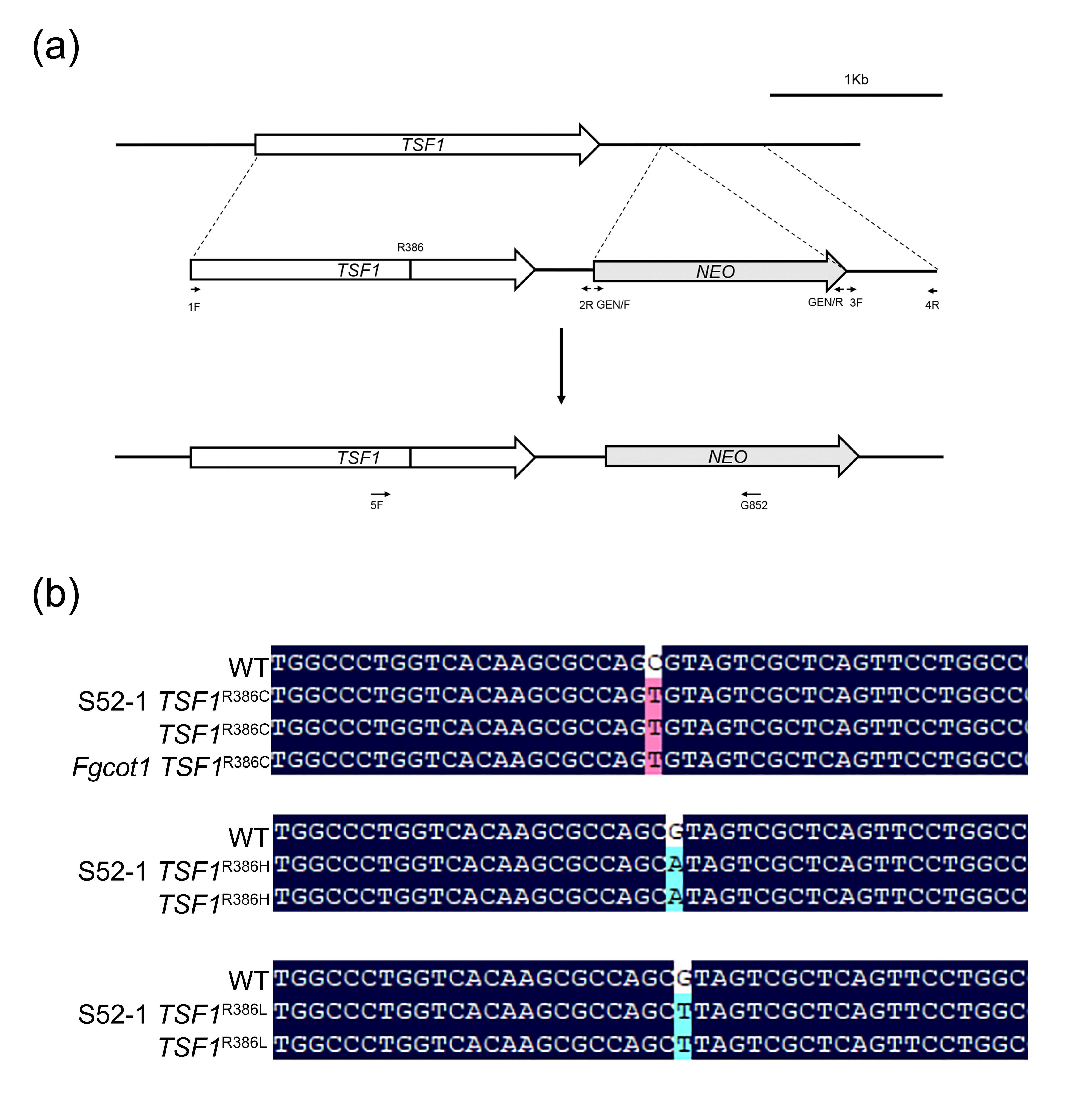

Supplement: Supplementary file 8 — Figure S8: Generation and sequence verification of the TSF1 R386C/H/L in situ mutations. (a) Schematic diagram of the primers used to introduce the point mutations. (b) Sequencing analysis confirming the S52 TSF1 R386C, TSF1 R386C, Fgcot1 TSF1 R386C, S52 TSF1 R386H, TSF1 R386H, S52 TSF1 R386L, TSF1 R386L mutants. [file MPP-27-e70321-s010.jpg]

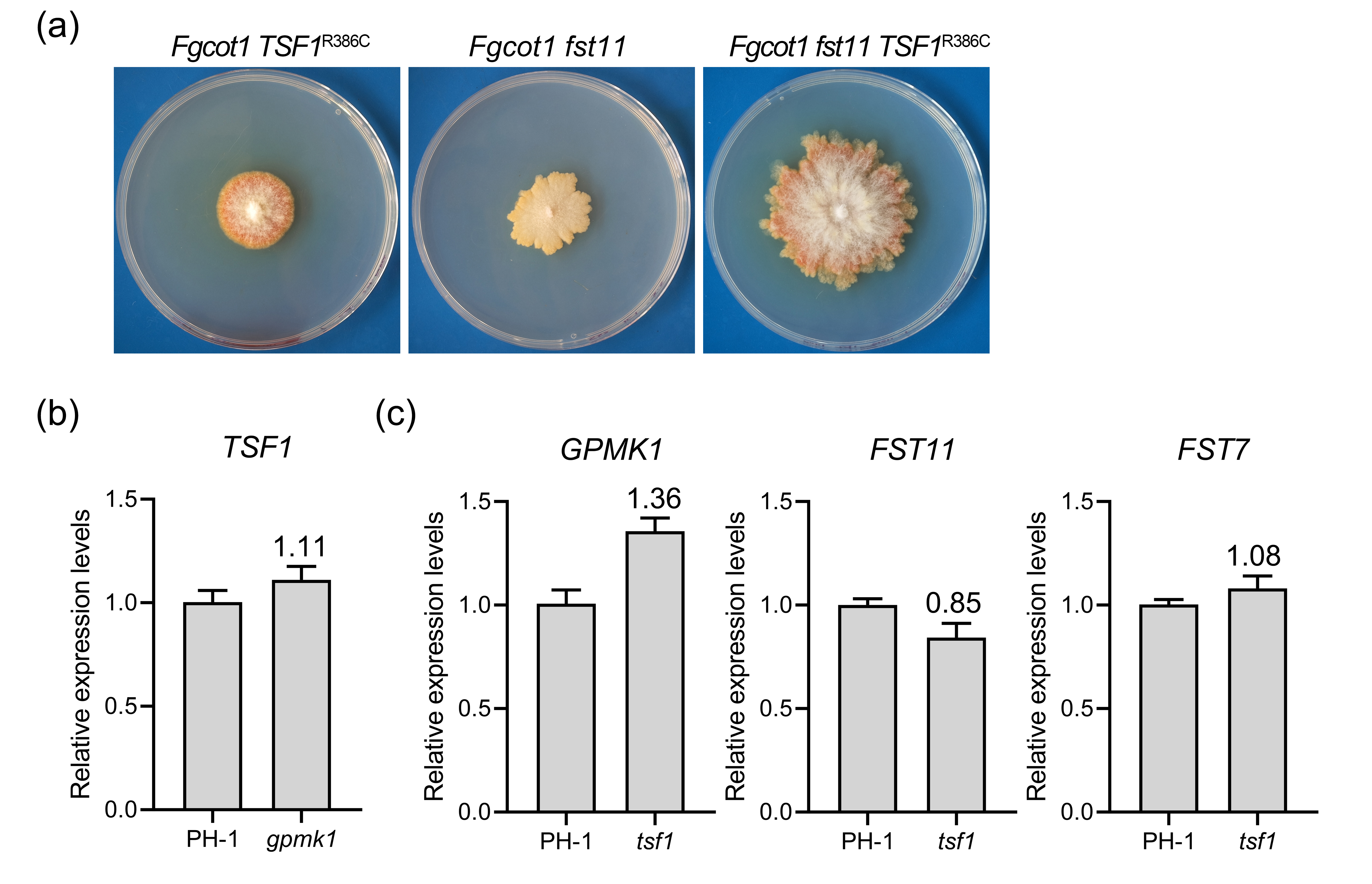

Supplement: Supplementary file 9 — Figure S9: The Fgcot1 fst11 TSF1 R386C triple mutant exhibits enhanced growth and no apparent transcriptional cross‐regulation between Tsf1 and the Gpmk1 pathway. (a) Colony morphology of the Fgcot1 TSF1 R386C, Fgcot1 fst11 and Fgcot1 fst11 TSF1 R386C mutants grown on complete medium (CM) for 10 days. (b). Relative expression of TSF1 in the gpmk1 mutant. (c). Relative expression of GPMK1, FST11 and FST7 in the tsf1 mutant. All expression changes remained within 1.4‐fold of wild‐type levels (0.71 < fold change < 1.4), indicating no apparent transcriptional cross‐regulation. [file MPP-27-e70321-s006.jpg]
