# Supplementary material for: FgCot1 Regulates Polarized Growth and Conidiation in Fusarium graminearum via Gpmk1 MAPK and Tsf1 Transcriptional Pathways
Source: Mol Plant Pathol. 2026 Jul 23;27(7):e70321. doi: 10.1111/mpp.70321 (PMC13396691; doi:10.1111/mpp.70321)
Supplement: Supplementary file 10 — Table S1: Polymerase chain reaction (PCR) primers used in this study. [file MPP-27-e70321-s012.docx]

**Table S1. Polymerase chain reaction (PCR) primers used in this study.**

| **Name** | **Sequence (5’-3’)** |
| --- | --- |
| COT1/1F | AAAACGACGAATGCTACCTG |
| COT1/2R | TTGACCTCCACTAGCTCCAGCCAAGCCCTGTTGTGCTGTGCTAAGGA |
| COT1/3F | GAATAGAGTAGATGCCGACCGCGGGTTTTGAGGAGTCCCCCGAGATG |
| COT1/4R | TTGGGTTCTGTTGATTATTATTGAC |
| COT1/5F | GACTGCCTATCCCACTACGC |
| COT1/6R | ATAAACCTTGCCATCTCCCTT |
| COT1/7F | TACATCTAAAGTGCAAGGTAGCC |
| COT1/8R | TAAAGACAGGCAGCATAAAGAGT |
| YG/F | GATGTAGGAGGGCGTGGATATGTCCT |
| HY/R | GTATTGACCGATTCCTTGCGGTCCGAA |
| HYG/F | GGCTTGGCTGGAGCTAGTGGAGGTCAA |
| HYG/R | AACCCGCGGTCGGCATCTACTCTATTC |
| H852 | ATGTTGGCGACCTCGTATTGG |
| H850 | TTCCTCCCTTTATTTCAGATTCAA |
| H855R | GCTGATCTGACCAGTTGC |
| H856F | GTCGATGCGACGCAATCGT |
| CM-COT1/F | CGACTCACTATAGGGCGAATTGGGTACTCAAATTGGCCGAGCAGCCTAGAGTGTCC |
| CM-COT1/R | CACCACCCCGGTGAACAGCTCCTCGCCCTTGCTCACATGACAGAGCGTGAGGAGCG |
| pFL2 insert/F | TAACGCCAGGGTTTTCCCAGTCA |
| pFL2 insert/R | CGTGCTGCTTCATGTGGTCGG |
| FST11/1F | GGTATTCGCTTTCCTTGCTG |
| FST11/2R | CAGATACGGCAGAGAAATCGCAACCTCGCAAGTCGCAGTGGGATAAC |
| FST11/3F | GTTTAGATTCCAAGTGTCTACTGCTGGCCGCCTAATGATTTGATTTGC |
| FST11/4R | TAGAGTGATAGAGTCTTCGGCG |
| FST11/5F | GTTGTCGCCAATCTCGTATC |
| FST11/6R | ATGCCCTTCTTACGATTGTC |
| FST11/7F | TATTCTTCTATGCGGCTCTG |
| FST11/8R | GACGGGAGTAGAGTGTGGAC |
| GEN/F | GAGGTTGCGATTTCTCTGCCGTATCTG |
| GEN/R | GCCAGCAGTAGACACTTGGAATCTAAAC |
| GE/R | CAGTCGATGAATCCAGAAAAGCG |
| EN/F | GGAAGGGACTGGCTGCTATTGG |
| G852 | TCGGCTATGACTGGGCACAACA |
| G850 | GAGCGGCGATACCGTAAAGCAC |
| G855/R | TGTTGGGTTTGAGCTAGGTGGG |
| G856/F | GAATGGTCAAATCAAACTGCTAGATAT |
| SSF1/1F | GAGCGAGCGTTTGGTTAGTTAT |
| SSF1/2R | CAGATACGGCAGAGAAATCGCAACCTCCCAAGTGGTGGAGGGAGGAG |
| SSF1/3F | GTTTAGATTCCAAGTGTCTACTGCTGGCCAGGCGTTTTTATTACAGCAAG |
| SSF1/4R | GGACCACATAGTCAACGGATTT |
| SSF1/5F | ATTGGGTAAGCGAATACAACACAT |
| SSF1/6R | TCCAGATGACCAGGAGGTAGATAG |
| SSF1/7F | TTGTGTGGGTTGAACTCGTCTATT |
| SSF1/8R | ATACTCTCTCCACACCATCCATCA |
| CM-SSF1/F | CGACTCACTATAGGGCGAATTGGGTACTCAAATTGGTGTCCAGTCCGCCCTGTCTA |
| CM-SSF1/R | CACCACCCCGGTGAACAGCTCCTCGCCCTTGCTCACAACGCTGTTCTCCTCGTCGTA |
| FST50Seq/F | CTCAGCCCATTCCATCTTGTG |
| FST50Seq/R | AGTATGACTTCACCATCGCCAAT |
| FST11Seq/F | CGTGCGTTACTAATAATCCCG |
| FST11Seq/R | ACCAAACTCCTTGTCCCTGAT |
| FST7Seq/F | TCAACTTTGCGGCTTCACTACC |
| FST7Seq/R | AGAGACCACGACAAAGGTTTCAAAA |
| FST12Seq/F | TGGTTGCGATTATCCAGACACTTC |
| FST12Seq/R | GACTACAAGGGATTTACGGCTTGAA |
| FST11_B/1F | CTGTCCGGTCTTTGTGTTGC |
| FST11_B/2R | ACCTCCACTAGCTCCAGCCAATTGGCTTGGAGGGGAATGG |
| FST11_B/3F | TGCTTGCAGGCATCTCATGATGAGACTCGGACGGGATTGA |
| FST11_B/4R | TGCTGCGGTGTCTTGTAGTT |
| FST11_B/5F | TCATTCGGCTGCGTTTACCT |
| FST11_B/6R | TCCGAGCGACCAAATATCCG |
| FST11_B/7F | TGAGGGGTGAGAGAGCTGAG |
| FST11_B/8R | TGGCTGTCTCCCTGTGAA |
| B850/F | TCAGCCCACTTGTAAGCAGTAGC |
| B852/R | CTGGATGCCGACGGATTTG |
| GPMK1 Seq/F | ACGCTCTCCGTTTTTTCTGACAA |
| GPMK1 Seq/R | TCCTGTTTCCTTACATTCAACCACC |
| SSF1Seq/F | CACACAAAGAAACGAAGCAACTCA |
| SSF1Seq/R | ACAATCCGTCCCTGAAAGCAATGA |
| TSF1site1F | CTCTGCGAACGGTGGTAA |
| TSF1site2R | CAGATACGGCAGAGAAATCGCAACCTC AAGAGATAAAACGAGATGCGG |
| TSF1site3F | GTTTAGATTCCAAGTGTCTACTGCTGGC GGGTGGTTTCTGTTGGTCA |
| TSF1site4R | ACCATCCGTAAGCCACCT |
| TSF1/1F | AAGTAGCTCCGTCAAGCCAC |
| TSF1/2R | TTGACCTCCACTAGCTCCAGCCAAGCCTGGCGGATTGGTTGGTAGTC |
| TSF1/3F | CGTCCGCAATGTGTTATTAAGTCGACATCGGTCATGTATGTCGGGC |
| TSF1/4R | TCACGGCAAGGACCAAAACT |
| TSF1/5F | ACGATACTTCATCCTTCCTGTTCT |
| TSF1/6R | TGGCCGTAGATGGGATGAGA |
| TSF1/7F | GAATGGCTCGGCTTTGGTC |
| TSF1/8R | GGGAGTCAACGCGAAAACAC |
| ADTSF1/F | GCCATGGAGGCCAGTGAATTCATGGCCACGGATATGGAT |
| ADTSF1/R | CCACTGCTTGGGTGGAATTCTTAGTTTCCGTGAGCATGG |
| BDCOT1/F | ATGGCCATGGAGGCCGAATTCATGGATCCCAACAACAGTAAC |
| BDCOT1/R | TCGACGGATCCCCGGGAATTCTTACCGGAAGTTGTTATCAAAC |
| pKNTG /TSF1F | AGGGAACAAAAGCTGGGTACCCTTTTGGACAGTGAGTGGG |
| pKNTG/TSF1-R | GAACAGCTCCTCGCCCTTGCTCACGTTTCCGTGAGCATGGG |
| pKNT-rp27-COT1-F | TTTCGTAGGAACCCAATCTTCAAAATGGATCCCAACAACAGTA |
| pKNT-rp27-COT1-R | GAACAGCTCCTCGCCCTTGCTCACCCGGAAGTTGTTATCAAACC |
| pKNT-RP27-TSF1-6HIS-F | AACCCAATCTTCAAACTCGAGATGGCCACGGATATGGATCAA |
| pKNT-RP27-TSF1-6HIS-R | TCAGTAACGTTAAGTGGATCCTTAATGGTGATGGTGATGATGGTTTCCGTGAGCATGGG |
| 4T-1-FST7/F | ATCTGGTTCCGCGTGGATCCATGTCCGATCCTTTCGCTCC |
| 4T-1-FST7/R | TCGAGTCGACCCGGGAATTCTCACTGTATGCCGTAGCTTGG |
| 4T-1-FST11/F | ATCTGGTTCCGCGTGGATCCATGGCCATGTTAGCTTCAAAAGC |
| 4T-1-FST11/R | TCGAGTCGACCCGGGAATTCCTACGTGATAGGGATCAAGAAGGG |
| 4T-1-TSF1/F | ATCTGGTTCCGCGTGGATCCATGGCCACGGATATGGATCAAT |
| 4T-1-TSF1/R | TCGAGTCGACCCGGGAATTCTTAGTTTCCGTGAGCATGGG |
| pCold-Cot1/F | TCGGTACCCTCGAGGGATCCATGGATCCCAACAACAGTAACAAT |
| pCold-Cot1/R | AGGTCGACAAGCTTGAATTCTTACCGGAAGTTGTTATCAAACCG |
