# Supplementary material for: FgCot1 Regulates Polarized Growth and Conidiation in Fusarium graminearum via Gpmk1 MAPK and Tsf1 Transcriptional Pathways
Source: Mol Plant Pathol. 2026 Jul 23;27(7):e70321. doi: 10.1111/mpp.70321 (PMC13396691; doi:10.1111/mpp.70321)
Supplement: Supplementary file 11 — Table S2: Relative growth rates of suppressor strains compared with the original Fgcot1 mutant. [file MPP-27-e70321-s013.docx]

**Table S2. Relative growth rates of suppressor strains compared with the original *Fgcot1* mutant.**

| **Suppressor strains** | **Growth increase ^a^** | **Suppressor strains** | **Growth increase ^a^** | **Suppressor strains** | **Growth increase ^a^** |
| --- | --- | --- | --- | --- | --- |
| S1 | 3.4±0.09 | S43 | 3.2±0.25 | S81 | 2.7±0.09 |
| S2 | 3.0±0.08 | S45 | 3.0±0.14 | S82 | 2.8±0.08 |
| S3 | 3.5±0.20 | S47 | 3.2±0.15 | S84 | 3.0±0.08 |
| S4 | 3.4±0.19 | S48 | 3.3±0.11 | S85 | 2.7±0.38 |
| S5 | 2.8±0.14 | S51 | 3.1±0.11 | S86 | 4.9±0.16 |
| S9 | 2.4±0.29 | S52 | 3.2±0.1 | S87 | 2.8±0.35 |
| S10 | 3.3±0.09 | S53 | 4.1±0.28 | S89 | 2.7±0.10 |
| S11 | 2.9±0.16 | S55 | 2.6±0.19 | S90 | 2.0±0.08 |
| S14 | 3.5±0.30 | S56 | 2.8±0.13 | S92 | 2.4±0.14 |
| S16 | 4.0±0.28 | S58 | 2.6±0.33 | S96 | 2.9±0.11 |
| S22 | 2.8±0.25 | S59 | 2.9±0.19 | S97 | 3.0±0.12 |
| S23 | 3.5±0.20 | S60 | 3.1±0.12 | S103 | 2.5±0.19 |
| S24 | 2.9±0.65 | S61 | 3.0±0.12 | S109 | 2.0±0.23 |
| S25 | 3.4±0.30 | S62 | 2.0±0.25 | S110 | 2.9±0.15 |
| S27 | 3.3±0.19 | S65 | 2.0±0.15 | S112 | 2.0±0.19 |
| S29 | 3.4±0.09 | S66 | 2.2±0.16 | S113 | 3.4±0.11 |
| S30 | 3.3±0.05 | S68 | 3.0±0.12 | S115 | 2.9±0.15 |
| S36 | 3.1±0.11 | S70 | 2.1±0.19 | S116 | 3.0±0.30 |
| S37 | 3.1±0.1 | S71 | 2.0±0.14 | S117 | 2.9±0.10 |
| S39 | 3.1±0.16 | S75 | 2.4±0.41 | S118 | 3.1±0.15 |
| S41 | 3.0±0.19 | S79 | 2.9±0.22 | S119 | 3.0±0.27 |

^a^, The growth rate of suppressor/the growth rate of *Fgcot1* mutant
