# Supplementary material for: FgCot1 Regulates Polarized Growth and Conidiation in Fusarium graminearum via Gpmk1 MAPK and Tsf1 Transcriptional Pathways
Source: Mol Plant Pathol. 2026 Jul 23;27(7):e70321. doi: 10.1111/mpp.70321 (PMC13396691; doi:10.1111/mpp.70321)
Supplement: Supplementary file 12 — Table S3: Candidate Gpmk1 pathway genes sequenced in the selected suppressor strains. [file MPP-27-e70321-s002.docx]

**Table S3. Candidate Gpmk1 pathway genes sequenced in the selected suppressor strains.**

| Fg | *FST50* | *FST11* | *FST7* | *GPMK1* | *FST12* |
| --- | --- | --- | --- | --- | --- |
| Sc | *STE50* | *STE11* | *STE7* | *KSS1/FUS3* | *STE12* |
| Mo | *MST50* | *MST11* | *MST7* | *PMK1* | *MST12* |
| S1 | - | Val836del | - | - | - |
| S4 | - | - | - | - | - |
| S5 | - | - | Asn193Ala | - | - |
| S10 | - | Ser593* | - | - | - |
| S16 | - | Leu285fs | - | - | - |
| S23 | - | Trp829* | - | - | - |
| S27 | - | - | Ser246fs | - | - |
| S29 | - | Arg329* | - | - | - |
| S36 | - | - | Glu231Gly | - | - |
| S45 | Met185fs | - | - | - | - |
| S48 | - | leu285fs | - | - | - |
| S52 | Arg174fs | - | - | - | - |
| S55 | Asn93fs | - | - | - | - |
| S59 | - | Gly807Ala | - | - | - |
| S60 | - | Trp626* | - | - | - |
| S62 | - | - | Gly235Asp | - | - |
| S75 | - | - | Gln321* | - | - |
| S85 | - | - | - | - | - |
| S89 | - | - | - | Arg74* | - |
| S90 | - | - | - | - | His78fs |
| S92 | - | Ser784Phe | - | - | - |
| S97 | - | Arg436* | - | - | - |
| S103 | - | - | - | Leu155del | - |
| S110 | - | - | Asp121fs | - | - |
| S115 | - | - | Cys309Tyr | - | - |

-, sequenced but no changes found

*, stop codon

fs, frameshift mutation
